# Recruitment of the Ulp2 protease to the inner kinetochore prevents its hyper-sumoylation to ensure accurate chromosome segregation

**DOI:** 10.1371/journal.pgen.1008477

**Published:** 2019-11-20

**Authors:** Raymond T. Suhandynata, Yun Quan, Yusheng Yang, Wei-Tsung Yuan, Claudio P. Albuquerque, Huilin Zhou

**Affiliations:** 1 Department of Cellular and Molecular Medicine, University of California at San Diego, La Jolla, California, United States of America; 2 Moores Cancer Center, University of California, San Diego, La Jolla, California, United States of America; Whitehead Institute, MIT, UNITED STATES

## Abstract

The kinetochore is the central molecular machine that drives chromosome segregation in all eukaryotes. Genetic studies have suggested that protein sumoylation plays a role in regulating the inner kinetochore; however, the mechanism remains elusive. Here, we show that *Saccharomyces cerevisiae* Ulp2, an evolutionarily conserved SUMO specific protease, contains a previously uncharacterized kinetochore-targeting motif that recruits Ulp2 to the kinetochore via the Ctf3^CENP-I^-Mcm16^CENP-H^-Mcm22^CENP-K^ complex (CMM). Once recruited, Ulp2 selectively targets multiple subunits of the kinetochore, specifically the Constitutive Centromere-Associated Network (CCAN), via its SUMO-interacting motif (SIM). Mutations that impair the kinetochore recruitment of Ulp2 or its binding to SUMO result in an elevated rate of chromosome loss, while mutations that affect both result in a synergistic increase of chromosome loss rate, hyper-sensitivity to DNA replication stress, along with a dramatic accumulation of hyper-sumoylated CCAN. Notably, sumoylation of CCAN occurs at the kinetochore and is perturbed by DNA replication stress. These results indicate that Ulp2 utilizes its dual substrate recognition to prevent hyper-sumoylation of CCAN, ensuring accurate chromosome segregation during cell division.

## Author summary

The kinetochore plays a central role in controlling accurate chromosome segregation in all eukaryotes. Defects in the kinetochore are a frequent cause of aneuploidy, which leads to birth defects and cancer in humans. Although the composition of the kinetochore has been extensively studied, how it is regulated remains poorly understood. Prior genetic studies have implicated an important role for protein sumoylation, the attachment of the Small Ubiquitin-like MOdifier (SUMO) to cellular proteins, in preventing aneuploidy. Here we identify a novel kinetochore-targeting motif in the SUMO specific protease Ulp2 and uncover the mechanism by which it selectively targets the inner kinetochore. We further show that the sumoylation level of the inner kinetochore is tightly regulated, and this regulation is necessary to prevent the aberrant loss of chromosomes during cell division. These findings uncover a critical molecular link between protein sumoylation and the suppression of aneuploidy.

## Introduction

The faithful transmission of genetic material during each cell division is vital for the survival of all living organisms. The kinetochore, a multi-subunit protein complex that assembles onto a specialized region of the chromatin called the centromere, is essential for proper chromosome segregation [1]. In *Saccharomyces cerevisiae*, kinetochores are assembled around a central histone H3 variant known as Cse4^CENP-A^, which marks the centromere. Kinetochore assembly is initiated by Mif2^CENP-C^, an evolutionarily conserved protein, which directs a hierarchical assembly of proteins to form the Constitutive Centromere-Associated Network (CCAN), whose structure has recently been determined [2–4]. Like most other kinetochore subunits, CCAN associates with the centromere throughout the cell cycle, and contains: Mif2, the Ctf19-Okp1-Mcm21-Ame1 (COMA) complex [5], Chl4-Iml3 [6], Ctf3-Mcm16-Mcm22 (CMM) [7] and Cnn1-Wip1-Mhf1/2 [8]; of these, Mif2 and Ame1-Okp1 play an early and essential role [9, 10]. Ctf19-Mcm21 directly binds to Ame1-Okp1 to form the COMA complex, followed by the sequential assembly of Chl4-Iml3, Ctf3-Mcm16-Mcm22 and Cnn1-Wip1. Moreover, mutants of the non-essential subunits of CCAN in *Saccharomyces cerevisiae* have been shown to impair faithful chromosome segregation [11–14]. Once fully assembled, CCAN provides a platform that recruits the outer kinetochore proteins, such as the KMN network (Knl1/Mis12 complex/Ndc80 complex), which connects the microtubules to facilitate chromosome movement [15, 16].

While much has been learned about the composition and assembly of the kinetochore, much less is known about how various post-translational modification pathways may regulate its activity [17]. An earlier genetic screen in the yeast *Saccharomyces cerevisiae* identified *SMT3* as a high-copy suppressor of a temperature-sensitive mutant of *mif2-3*, an essential protein required for proper chromosome segregation [18]. *SMT3* encodes the yeast **S**mall **U**biquitin-like **MO**difier (**SUMO**), which similarly suppresses the temperature-sensitivity of the CENP-C^Mif2^ mutant in chicken cells [19]. SUMO is a member of the ubiquitin-like protein family, and is highly conserved throughout all eukaryotes [20]. Sumoylation, like ubiquitination, utilizes a three-step enzymatic cascade to modify target proteins, which begins with an E1 activating enzyme (Aos1-Uba2 in *S*. *cerevisiae*), followed by an E2 conjugating enzyme (Ubc9) and ends with several E3 ligases (Siz1, Siz2 and Mms21 in *S*. *cerevisiae*), which catalyze the covalent attachment of SUMO to its target proteins [21]. Sumoylation, being a highly dynamic and reversible post-translational modification, is removed from its target proteins by a group of enzymes known as the Ulp/SENP (ubiquitin-like protease/sentrin-specific protease) family of proteases, which cleave the isopeptide bond between SUMO and its substrate. The founding members of the Ulp/SENP family of enzymes are *S*. *cerevisiae* Ulp1 and Ulp2 [22, 23], whereas six SENPs, SENP1–3 and SENP5–7, have been found in humans [24]. Interestingly, *SMT4*, another suppressor of *mif2-3* [18], was found to encode the Ulp2 protease [23, 25]. This suggests that homeostasis of intracellular sumoylation, possibly at the kinetochore, may be needed to suppress the conditional lethality of the *mif2-3* mutant. Moreover, the *ulp2Δ* mutant exhibits defects in mitotic progression and chromosome segregation [23, 25, 26], and was recently reported to specifically accumulate aneuploidy of chromosome I [27]; this further suggests an important role for Ulp2 in maintaining proper chromosome segregation. The mechanism by which Ulp2 promotes proper chromosome segregation appears to be conserved; mutation of Ulp-4, an ortholog of Ulp2 in *C*. *elegans*, results in a chromosome segregation defect [28], while the knockdown of SENP6, the human ortholog of Ulp2, also affects chromosome segregation and causes mis-localization of the inner kinetochore complex CENP-H/I/K [29]. Altogether, these findings suggest an important role for Ulp2^SENP6^, and ultimately a role for SUMO homeostasis, in maintaining proper chromosome segregation, possibly via Mif2^CENP-C^ mediated kinetochore assembly.

We previously identified the substrates of Ulp2 via a proteome-wide approach, showing that Ulp2 specifically targets protein complexes at three distinct chromosomal regions, which include the nucleolar RENT complex, the MCM complex and the inner kinetochore CCAN complex [30]. Remarkably, the loss of Ulp2 was found to increase CCAN sumoylation by nearly 20-fold, including Mcm21, Mcm16, Mcm22, Ame1 and Okp1, indicating that Ulp2 specifically targets CCAN [30]. This was followed by two studies, which together demonstrated that Ulp2 targets its nucleolar substrates via a dual-substrate recognition mechanism [31, 32]; Ulp2 localizes to the nucleolus through its binding to the Csm1 nucleolar protein, where it then specifically targets poly-sumoylated substrates through its C-terminal SUMO-interacting motif (SIM). Interestingly, a mutation in Ulp2’s SIM resulted in a synergistic growth defect when it was combined with a mutation in a C-terminal Conserved Region (CCR) of Ulp2 (a.a. 931–934), whose function had not been defined [31]. Here we characterize this CCR of Ulp2 as a kinetochore-targeting motif that recruits Ulp2 to the kinetochore by directly binding to the Ctf3^CENP-I^-Mcm16^CENP-H^-Mcm22^CENP-K^ (CMM) complex. Ulp2 then utilizes its SIM to target hyper-sumoylated CCAN subunits, preserving the SUMO homeostasis of the kinetochore machinery. Thus, Ulp2 promotes accurate chromosome segregation through the tight regulation of CCAN sumoylation to maintain SUMO homeostasis of the kinetochore.

## Results

### Ulp2’s CCR and SIM are both required for maintaining CCAN sumoylation in *HF-SMT3* cells

**Fig 1:**
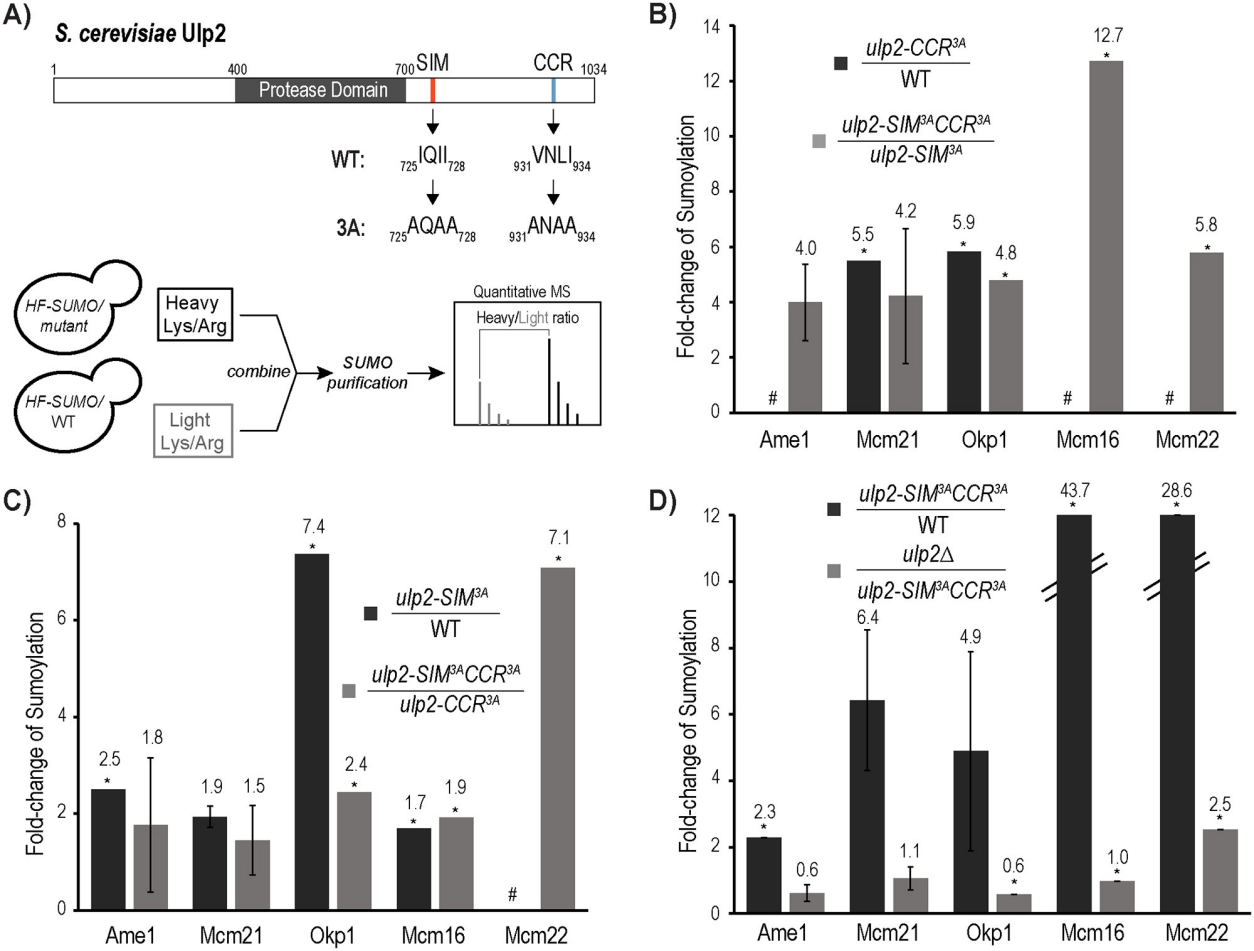
The C-terminal Conserved Region (CCR) and SUMO-interacting motif (SIM) of Ulp2 are both needed to desumoylate the CCAN complex. A) Illustration depicting Ulp2’s domain structure and the quantitative MS approach used to determine the effect of various *ulp2* mutations on sumoylated CCAN in the *HF-SMT3* (6×His-3×FLAG-Smt3) strain background (see experimental methods for details). Conserved hydrophobic residues in Ulp2’s SIM and CCR are indicated. Triple alanine mutations of these residues generate the *ulp2-SIM*^*3A*^ and *ulp2-CCR*^*3A*^ mutants. B-D) Effects of various *ulp2* mutations on sumoylated CCAN subunits are shown, while the rest of the MS results can be found in S1–S6 Tables. In each case, the fold-changes in the sumoylated CCAN subunit are shown for each of the indicated strains. CCAN subunits not identified in the MS experiments are indicated by #. Asterisks (*) indicate where an insufficient number of peptides are available for statistical analysis.

In our prior study, we showed that the *ulp2-781Δ* mutant, but not the *csm1Δ* mutant, elevates the sumoylation of both the inner kinetochore and nucleolar proteins [32]. This suggests that in addition to Csm1-binding, Ulp2^781-1034^ contains additional sequence elements that direct Ulp2 to desumoylate CCAN. Sequence alignments of Ulp2’s fungal orthologs revealed three conserved hydrophobic residues in the CCR of Ulp2 (a.a. 931–934) (Fig 1A, upper panel), which, when mutated to alanine and combined with the *ulp2-SIM*^*3A*^ mutation, resulted in a synergistic growth defect [31]. To determine the effect of these *ulp2* mutations on kinetochore sumoylation, we used the *HF-SMT3* (*6×His-3×Flag-SMT3*) strain and the quantitative SUMO proteomic method developed in our previous studies [30, 33] (Fig 1A, lower panel). We found that the *ulp2-CCR*^*3A*^ mutation results in an almost five-fold increase in the amount of sumoylated Mcm21 and Okp1 (Fig 1B), while the sumoylation of Ulp2’s substrates in the nucleolus and at origins of DNA replication are not appreciably affected (S1 Table). Because sumoylated CCAN exists at a low level in wild-type cells that is often below the MS detection limit, we next compared CCAN sumoylation levels in the *ulp2- SIM*^*3A*^*CCR*^*3A*^ double mutant to its levels in the *ulp2-SIM*^*3A*^ mutant, where sumoylated Ulp2 substrates accumulate to higher levels [31]. We found that the *ulp2-CCR*^*3A*^ mutant causes CCAN sumoylation to increase by over four-fold in the *ulp2-SIM*^*3A*^ strain background (Fig 1B and S2 Table), similar to its effect in the wild-type background.

We next examined the effect of the *ulp2-SIM*^*3A*^ mutation and found that it caused a moderate increase in the amount of sumoylated Ame1, Mcm21, Okp1 and Mcm16, indicating a role for Ulp2’s SIM in facilitating CCAN desumoylation by Ulp2 (Fig 1C, S3 Table). Similarly, this effect is also observed in the *ulp2-CCR*^*3A*^ background, as sumoylated CCAN subunits, including Ame1, Mcm21, Okp1, Mcm16 and Mcm22, accumulated to higher levels in the *ulp2-SIM*^*3A*^*CCR*^*3A*^ double mutant compared to the *ulp2-CCR*^*3A*^ single mutant (Fig 1C, S4 Table). In fact, the *ulp2-SIM*^*3A*^*CCR*^*3A*^ double mutant drastically increases the sumoylation of multiple CCAN subunits compared to the wild-type strain (Fig 1D, S5 Table). Moreover, sumoylation of CCAN reaches a level that phenocopies that of the *ulp2Δ* mutant, indicating that Ulp2’s CCR and SIM play a partially redundant role in directing Ulp2 to desumoylate CCAN (Fig 1D, S6 Table).

### CCAN abundance is not appreciably affected in the *ulp2-CCR*^*3A*^ and *ulp2-SIM*^*3A*^ mutants

**Fig 2:**
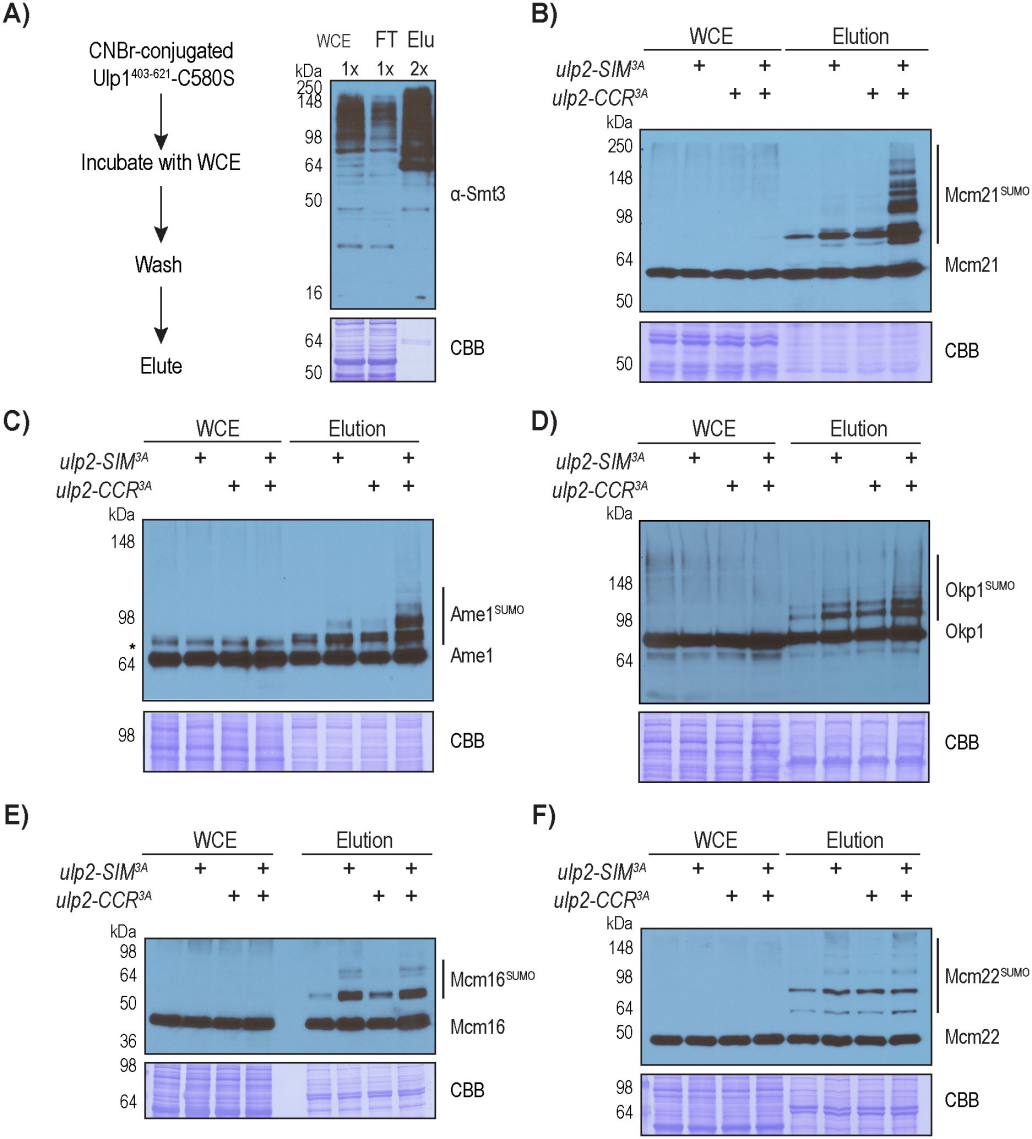
Desumoylation of CCAN is partially redundantly controlled by Ulp2’s SIM and CCR. A) Anti-Smt3 Western blot to observe total sumoylated proteins via the Ulp1-C580S pull-down approach. Relative loading amounts are indicated at the top of each sample lane. 2B-2F) Protein A tagged Mcm21, Ame1, Okp1, Mcm16 and Mcm22 were detected using an anti-Protein A antibody and Western blot analysis. Approximately 150-fold more loading was used for the eluted samples compared to the input samples. Sumoylated species of these CCAN subunits were only detected in the purified sample, which appear as higher molecular weight bands compared to the corresponding un-sumoylated CCAN subunit.

The strains used in the above proteomic approach made use of HF-Smt3, which allows for the enrichment of total sumoylated proteins, but the HF tag on Smt3 compromises poly-sumoylation to an unknown extent [30, 33]. A previous study showed that poly-sumoylated protein could be targeted for degradation, which was perturbed by the HF tag on Smt3 [32]. Additionally, our proteomic approach specifically measures the relative change in the amount of proteins that are conjugated to SUMO; thus the poly-sumoylation status or the protein levels of CCAN subunits could not be determined. To address these concerns, we took advantage of Ulp1-C580S, a catalytically inactive form of Ulp1, which exhibits a strong affinity to SUMO itself [34, 35]. We first immobilized recombinant Ulp1^403-621^-C580S onto CNBr-activated resin, and then tested its ability to purify endogenous sumoylated proteins from yeast cell extracts, which resulted in the recovery of approximately 50% of SUMO conjugates (Fig 2A). To facilitate the detection of CCAN in the *ulp2-CCR*^*3A*^, *ulp2-SIM*^*3A*^ and *ulp2-SIM*^*3A*^*CCR*^*3A*^ mutants, an endogenous Protein A tag was fused to the C-terminus of each CCAN subunit (Ame1, Mcm21, Okp1, Mcm16 and Mcm22). As shown in Fig 2B, the expression level of Mcm21 was unaffected by any of the *ulp2* mutations. Furthermore, a higher molecular weight species of Mcm21 is enriched in the elution, and is referred to as sumoylated Mcm21. Both the *ulp2-SIM*^*3A*^ and *ulp2-CCR*^*3A*^ single mutants caused a modest but appreciable increase in the abundance of sumoylated Mcm21, while the *ulp2-SIM*^*3A*^*CCR*^*3A*^ double mutant caused a drastic accumulation in the levels and species of sumoylated Mcm21 (Fig 2B), demonstrating partially redundant roles for Ulp2’s SIM and CCR in suppressing excessive Mcm21 sumoylation.

Similar to Mcm21, several species of sumoylated Ame1 and Okp1 were detected in wild type cells, which are modestly elevated in the *ulp2-SIM*^*3A*^ and *ulp2-CCR*^*3A*^ mutants, but more drastically accumulate in the *ulp2-SIM*^*3A*^*CCR*^*3A*^ mutant (Fig 2C and 2D). A slower migrating species of Ame1 was detected in cell lysate, but was determined to be unrelated to SUMO, as it is insensitive to Ulp1 cleavage (S1 Fig). However, the higher molecular weight species of Ame1 is referred to as sumoylated Ame1, as it is enriched via the Ulp1-C580S resin and can be cleaved by Ulp1 (S1 Fig). Unlike the COMA subunits (Ame1, Okp1 and Mcm21), the levels of sumoylated Mcm16 and Mcm22 increase modestly in the *ulp2-CCR*^*3A*^ mutant, but in the *ulp2-SIM*^*3A*^ mutant they accumulate to a higher level comparable to the level in the *ulp2-SIM*^*3A*^*CCR*^*3A*^ double mutant (Fig 2E and 2F). This behavior differs from the MS findings above for these proteins, which could be attributed to the fact that the untagged WT Smt3 strain was used here. For example, hyper-sumoylated Mcm16 and Mcm22 could have already reached a maximum level in the *ulp2-SIM*^*3A*^ mutant, which prevents the detection of any additional effect from *ulp2-CCR*^*3A*^. We also did not investigate the other CCAN subunits Ctf3 and Ctf19, whose sumoylation was not detected by MS possibly due to their lower levels. In all cases, the protein levels of unmodified CCAN subunits are not grossly altered in any of the *ulp2* mutants, suggesting that these effects are not due to a change in CCAN expression. Overall, the findings here are in general agreement with the MS results performed using HF-Smt3 cells (Fig 1), despite accurate quantification of sumoylated CCAN by MS was limited by their low abundance, especially in wild type cells.

### The CMM complex mediates the association of Ulp2 to the kinetochore

**Fig 3:**
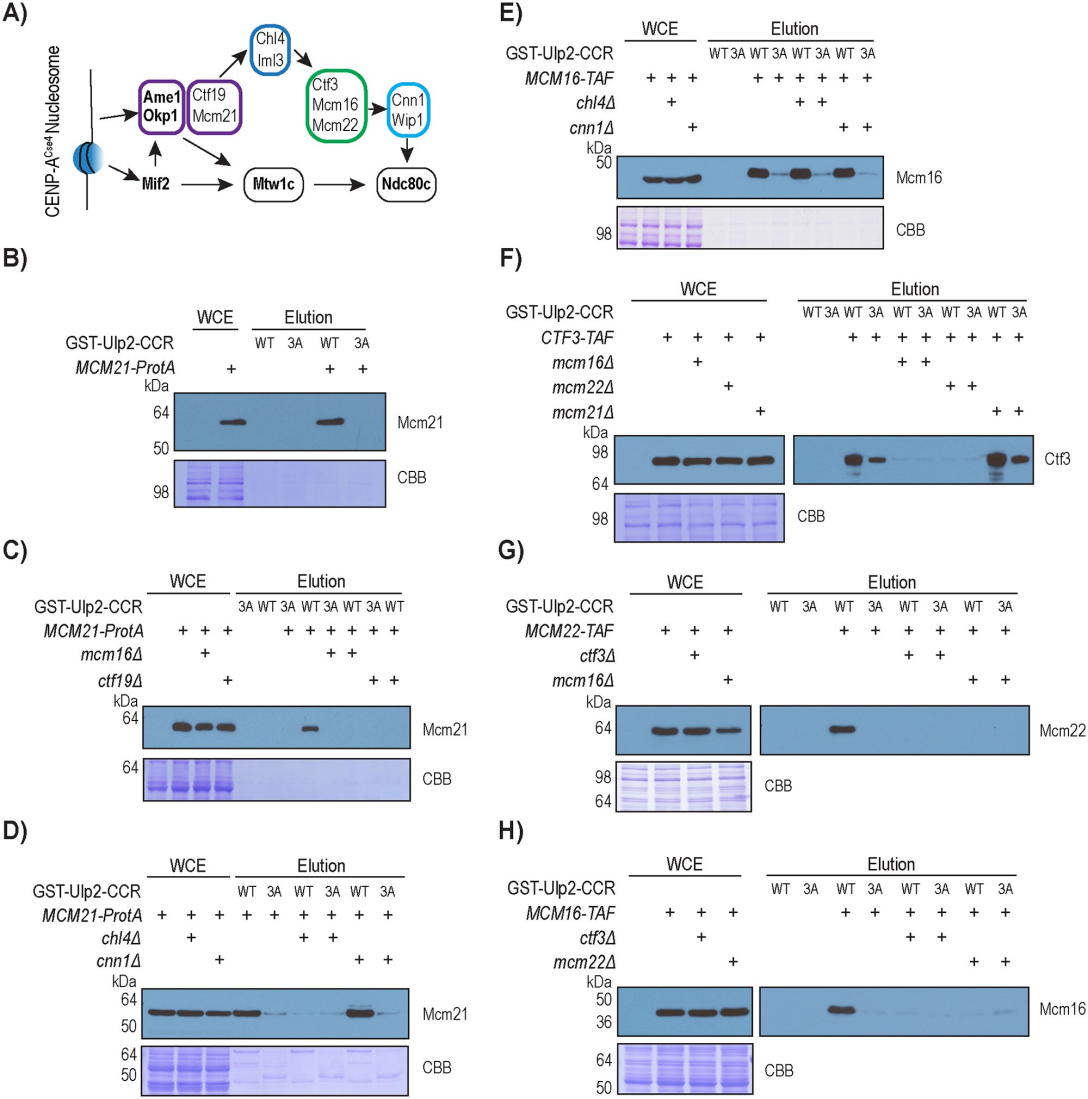
Binding between Ulp2’s CCR and the kinetochore requires the Ctf3-Mcm16-Mcm22 complex. A) Illustration of the hierarchical order of CCAN assembly. Various sub-complexes of CCAN are distinguished in colored boxes, in which essential subunits are shown in ***bold***. B) Pull-down assay to observe the binding between Mcm21 and Ulp2’s CCR. C) Effect of *mcm16Δ* and *ctf19Δ* on the binding between Mcm21 and Ulp2’s CCR. D) Effect of *chl4Δ* and *cnn1Δ* on the binding between Mcm21 and Ulp2’s CCR. E) Effect of *chl4Δ* and *cnn1Δ* on the binding between Mcm16 Ulp2’s CCR. F) Effect of *mcm16Δ*, *mcm22Δ* and *mcm21Δ* on the binding between Ctf3 and Ulp2’s CCR. G) Effect of *mcm16Δ* and *ctf3Δ* on the binding between Mcm22 and Ulp2’s CCR. H) Effect of *mcm22Δ* and *ctf3Δ* on the binding between Mcm16 and Ulp2’s CCR. TAF tag refers to 6xHIS-3xFLAG-ProteinA (see footnote in S12 Table).

Prior studies have demonstrated that CCAN assembles at centromeres in a hierarchical order (Fig 3A) [9, 10, 36], and considering that Ulp2’s CCR seems to play a specific role in desumoylating CCAN, we tested whether Ulp2 binds to the kinetochore through its CCR. Using an immobilized Ulp2^873-1034^ (Ulp2-CCR) affinity column (S1 Fig), we found that the wild-type Ulp2-CCR resin binds to Mcm21 in yeast cell extracts, and that this binding is abolished by the *ulp2-CCR*^*3A*^ mutation (Fig 3B). Interestingly, deletion of Ctf19 or Mcm16 also abolished the binding between Ulp2 and Mcm21 without affecting the abundance of Mcm21 (Fig 3C), indicating that Mcm21 does not directly bind to Ulp2. Because Mcm16 is required for the Ulp2-Mcm21 interaction, we next investigated whether deleting Chl4 and Cnn1 had any effect. Deletion of Cnn1, which acts downstream of Mcm16 in CCAN assembly, did not disrupt the binding between Ulp2 and Mcm21, whereas the deletion of Chl4, which helps to recruit Mcm16 to Mcm21, abolished the binding (Fig 3D). In contrast, deletion of Chl4 or Cnn1 did not disrupt binding between Ulp2’s CCR and Mcm16 (Fig 3E), further implying that Mcm16 may directly interact with Ulp2. Moreover, the deletion of any individual subunit of CMM (Ctf3-Mcm16-Mcm21) eliminated Ulp2’s interaction with the other two CMM subunits (Fig 3F–3H), indicating that an intact CMM complex is needed to interact with Ulp2. In all cases, the *ulp2-CCR*^*3A*^ mutation reduced or abolished its binding to CMM, confirming the specificity of this interaction. Taken together, these findings suggest that Ulp2 interacts with the CMM complex, which in turn mediates Ulp2’s interaction with the other CCAN sub-complexes. How Ulp2 may interact with the fully assembled kinetochore remains to be determined.

### Ulp2’s CCR is sufficient for its interaction with the CMM complex

**Fig 4:**
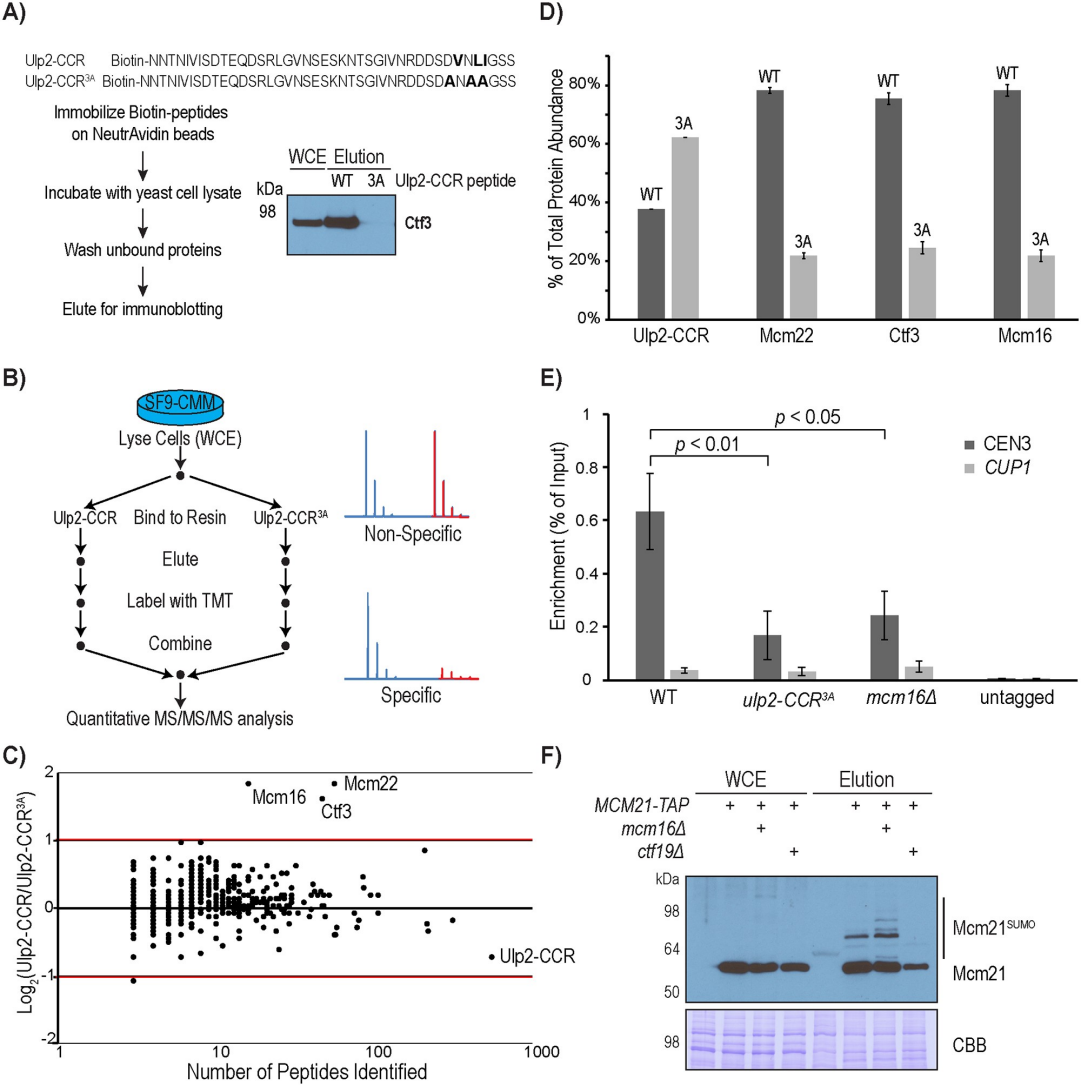
Ulp2’s CCR directly binds to the CMM complex. A) Experimental schematic and Western blot analysis for detecting the binding of a synthetic Ulp2-CCR peptide with Ctf3 in yeast cell extract. B) Experimental design to use TMT-based quantitative MS to compare the binding proteins of Ulp2’s CCR, purified using SF9 cells expressing the CMM complex. C) Log_2_ ratios of proteins associating with Ulp2-CCR versus Ulp2-CCR^3A^, identified by MS, are plotted on the Y-axis, while the number of peptides identified for each protein is plotted on the X-axis. D) Quantification of the relative abundance of Mcm16, Ctf3, Mcm22 and Ulp2-CCR is shown in Figure 4C. Error bars were calculated based on the standard error of the mean of TMT reporter ions found in multiple peptides of each protein. E) ChIP-qPCR analysis to measure the association of Ulp2 to the Centromere-III revealed a partial role for Ulp2’s CCR and Mcm16. The *p*-values indicate statistically significant differences of Ulp2-TAF with Ulp2-CCR^3A^-TAF and *mcm16Δ* using a two-tailed Student’s t-test. F) Effect of *mcm16Δ* and *ctf19Δ* on sumoylated Mcm21, which was purified using the Ulp1-C580S affinity resin (see Fig 2).

Considering the role of the conserved motif (a.a. 931–934) in Ulp2’s CCR in mediating the interaction between Ulp2 and CMM (Fig 3), we next sought to determine the minimal region in Ulp2 that is sufficient for binding to CMM. A synthetic peptide containing the conserved motif (Ulp2^896-937^) was found to bind to Ctf3 in yeast cell lysates, while a synthetic peptide (Ulp2^896-937^) containing the *CCR*^*3A*^ mutation does not bind (Fig 4A), confirming the specificity of this interaction. To further determine whether the binding between Ulp2 and CMM is direct, we expressed the CMM complex in SF9 insect cells, eliminating any interference from other yeast proteins, and used Tandem Mass Tag (TMT) based quantitative mass spectrometry (MS) to analyze specific proteins that bound to resins immobilized with either Ulp2-CCR or Ulp2-CCR^3A^ peptides (Fig 4B). As shown in Fig 4C and S7 Table, Ctf3, Mcm16 and Mcm22 of the CMM complex were the only proteins that specifically bind to the Ulp2-CCR peptide resin. In fact, Ctf3, Mcm16 and Mcm22 were found to be more than three-fold enriched by the wild-type Ulp2-CCR peptide resin compared to the Ulp2-CCR^3A^ peptide resin (Fig 4D), even though more Ulp2-CCR^3A^ peptide resin was used for the pull-down. Taken together, these results demonstrate that Ulp2’s CCR directly binds to the CMM complex.

To address whether the interaction between Ulp2 and CMM plays a role in recruiting Ulp2 to the kinetochore assembled at the centromere, chromatin immunoprecipitation and quantitative PCR (ChIP-qPCR) were performed to measure the amount of DNA bound to endogenously tagged Ulp2. This reveals that Ulp2 specifically binds to the centromere-III region but not the *CUP1* region (Fig 4E). This centromere-specific binding of Ulp2 is partially reduced by *ulp2-CCR*^*3A*^, similar to that caused by *mcm16Δ* (Fig 4E). This partial effect is in agreement with the partial role of Ulp2’s CCR in facilitating CCAN desumoylation (Figs 1 and 2), and it further suggests that Ulp2’s SIM and protease domain could be responsible for the remaining centromere binding activity. Consistent with the role of Mcm16 in recruiting Ulp2 to the centromere, deletion of Mcm16 led to an elevated level of sumoylated Mcm21 (Fig 4F), while deletion of Ctf19, which disrupts the formation of the Ctf19-Mcm21 sub-complex [5], caused a complete loss of sumoylated Mcm21. Thus, localization of Ulp2 to the centromere via Mcm16 plays a role in keeping the sumoylation of Mcm21 low.

### The CMM complex plays two roles in maintaining SUMO homeostasis of the inner kinetochore

**Fig 5:**
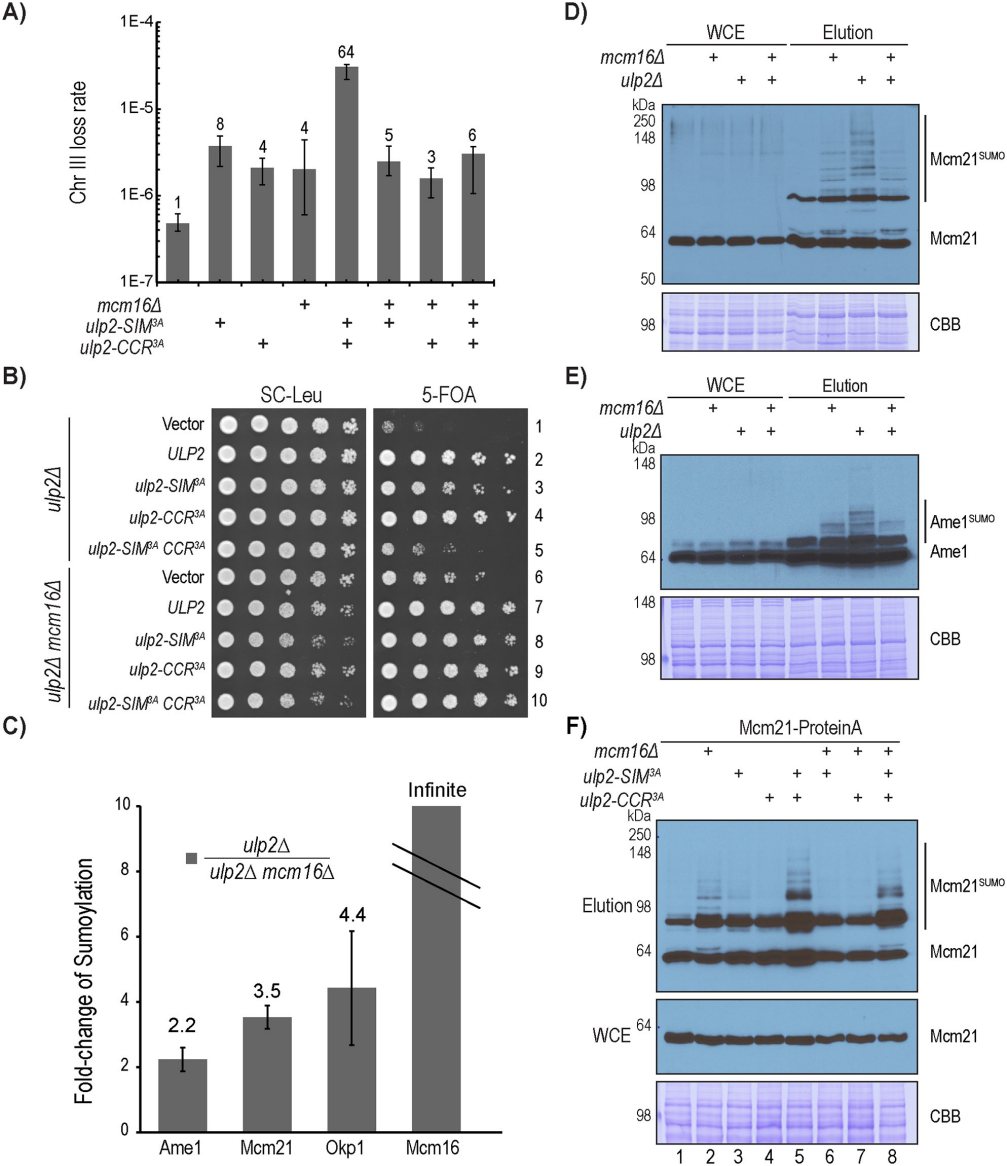
Mcm16 plays a dual role in regulating CCAN sumoylation. A) Chromosome loss rates of WT, *ulp2-SIM*^*3A*^, *ulp2-CCR*^*3A*^, *ulp2-SIM*^*3A*^*CCR*^*3A*^, *mcm16Δ*, *mcm16Δ ulp2-SIM*^*3A*^, *mcm16Δ ulp2-CCR*^*3A*^ and *mcm16Δ ulp2-SIM*^*3A*^*CCR*^*3A*^ mutants, measured by quantitative mating (also see S8 Table). B) Growth of various *ulp2* and *mcm16Δ* mutants, following the acute removal of the complementing *ULP2* plasmid by 5-fluoroorotic acid (5-FOA). C) Quantitative MS to measure the effect of *mcm16Δ* on intracellular sumoylation in the *ulp2Δ* mutant (also see S9 Table). D-E) Western blot analysis to observe the effect of *mcm16Δ* on Mcm21 and Ame1 sumoylation in the *ulp2Δ* mutant, following the enrichment of sumoylated proteins via the Ulp1-C580S affinity resin. F) Western blot analysis to observe the effect of *mcm16Δ* on the sumoylation of Mcm21 in various *ulp2* mutants following the enrichment of sumoylated proteins via Ulp1-C580S affinity resin.

Considering the biochemical defects of the *ulp2* mutants in CCAN binding and desumoylation, we next evaluated its potential defect in chromosome segregation by performing a quantitative mating assay to measure the rate of chromosome-III loss in the *ulp2* mutants [37]. As seen in Fig 5A and S8 Table, compared to wild-type cells, the *ulp2-SIM*^*3A*^ and *ulp2-CCR*^*3A*^ mutations result in an 8-fold and 4-fold respective increase in the rate of chromosome-III loss, while the *ulp2-SIM*^*3A*^*CCR*^*3A*^ double mutant causes a synergistic 64-fold increase in the rate of chromosome loss. These results indicate that Ulp2’s CCR and SIM play a partially redundant role in preventing chromosome loss.

Given that CMM directly binds to Ulp2 (Fig 4), we next asked whether a mutation in this complex would result in a chromosome loss defect similar to that of the *ulp2-CCR*^*3A*^, and further cause a synergistic increase in chromosome loss when it is combined with the *ulp2-SIM*^*3A*^. To address this, a quantitative mating assay was performed, revealing that the *mcm16Δ* mutant caused a 4-fold increase in the rate of chromosome loss (Fig 5A and S8 Table). Surprisingly, the *mcm16Δ* mutant did not further increase the rate of chromosome loss when combined with *ulp2-SIM*^*3A*^; instead, loss of Mcm16 in the *ulp2-SIM*^*3A*^*CCR*^*3A*^ mutant drastically reduced the rate of chromosome loss from 64-fold to 6-fold. To explore this further, we examined the effect of *mcm16Δ* on the growth of various *ulp2* mutants. As in our previous work [31], mutations to Ulp2’s SIM and CCR result in a growth defect when the complementing wild-type Ulp2 is acutely removed by plasmid shuffling (Fig 5B, rows 2–5). Interestingly, *mcm16Δ* strongly suppressed both the growth defect of the *ulp2Δ* mutant (Fig 5, rows 1 and 6) and that of the *ulp2-SIM*^*3A*^*CCR*^*3A*^ mutant (Fig 5, rows 5 and 10). These findings raise the possibility that Mcm16 plays another role that is distinct from recruiting Ulp2 (Figs 3 and 4).

To explore other functions of Mcm16, we performed quantitative MS in the *ulp2Δ* background to determine whether the loss of Mcm16 has any effect on sumoylated CCAN. As seen in Fig 5C and S9 Table, we observed approximately 2–4 folds more sumoylated Ame1, Mcm21 and Okp1 in the *ulp2Δ* single mutant than the *ulp2Δ mcm16Δ* double mutant, besides the expected absence of sumoylated Mcm16 in the *ulp2Δ mcm16Δ* double mutant. To confirm this, we purified total sumoylated proteins using the Ulp1-C580S pull-down method (Fig 2A). As shown in Fig 5D and 5E, *mcm16Δ* reduced the hyper-sumoylated species of Mcm21 and Ame1 in the *ulp2Δ* mutant background, whereas the amount of sumoylated Mcm21 and Ame1 was increased in the wild-type *ULP2* background. Moreover, *mcm16Δ* caused a modest reduction of hyper-sumoylated Mcm21 in the *ulp2-SIM*^*3A*^*CCR*^*3A*^ double mutant (Fig 5F, lanes 5 and 8), although little effect was observed in the *ulp2* single mutants. Overall, these findings indicate that Mcm16 plays two opposing roles in regulating kinetochore sumoylation: 1) recruiting Ulp2 to desumoylate Mcm21 and Ame1, and 2) facilitating the sumoylation of Mcm21 and Ame1, which is more clearly observed in cells lacking Ulp2. It should be noted that these opposing roles of Mcm16 are not contradictory to each other; instead, they suggest that separation-of-function mutations in the CMM complex would be needed to understand these roles further.

### The homeostasis of CCAN sumoylation is critical for accurate chromosome segregation

**Fig 6:**
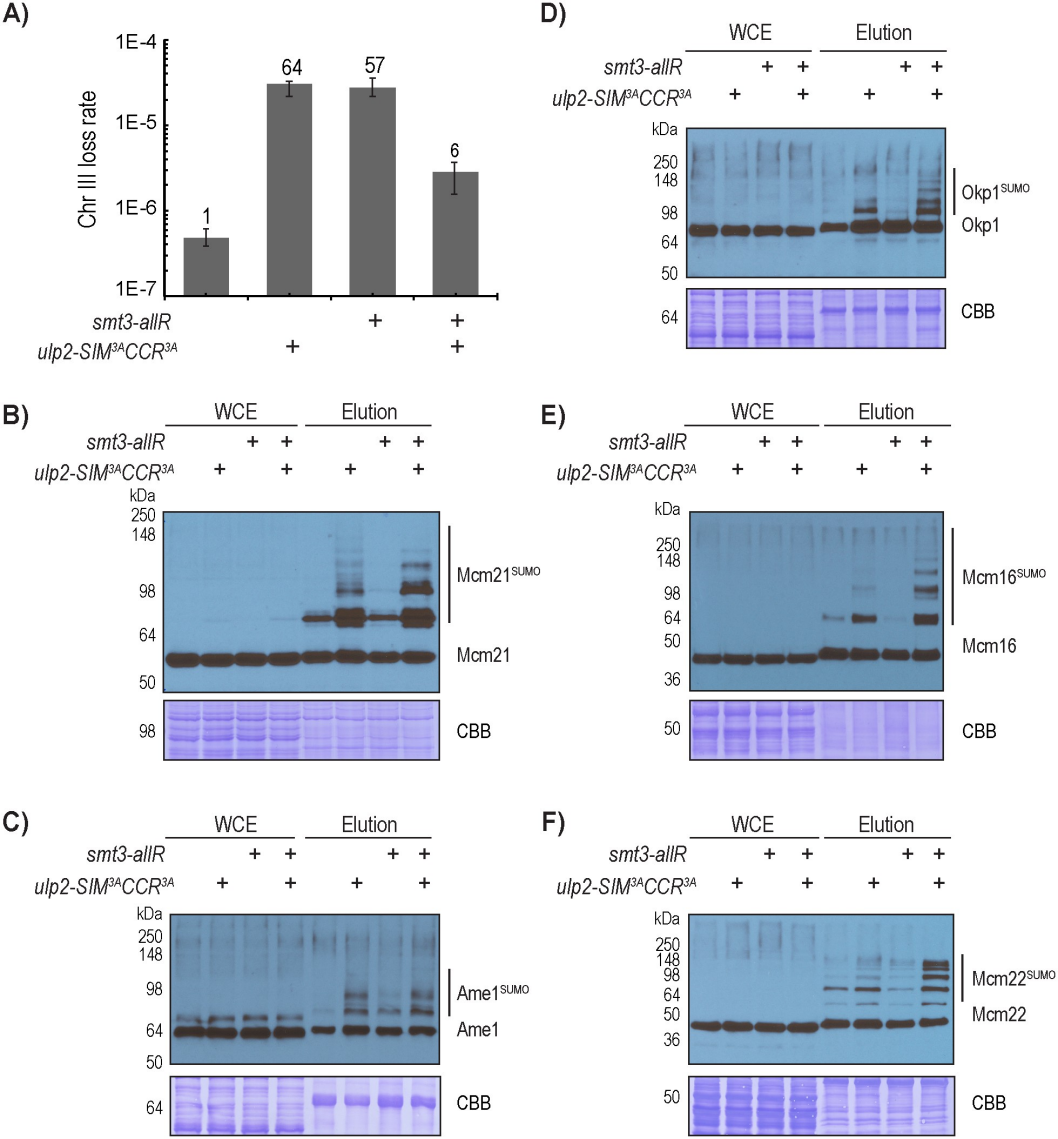
CCAN sumoylation and chromosome segregation are affected by changes in poly-sumoylation. A) Chromosome loss rates, measured by quantitative mating, of WT, *smt3-allR*, *ulp2-SIM*^*3A*^*CCR*^*3A*^, and *smt3-allR ulp2-SIM*^*3A*^*CCR*^*3A*^ triple mutants (also see S8 Table). B-F) The effects of *smt3-allR* on the sumoylation patterns of CCAN subunits in wild-type *ULP2* and the *ulp2-SIM*^*3A*^*CCR*^*3A*^ mutant backgrounds.

Our results suggest that an accumulation of hyper-sumoylated CCAN leads to growth defects and elevated chromosome loss in the *ulp2* mutants (Fig 5). If so, the *smt3-allR* mutant, in which formation of poly-SUMO chains is blocked [38], should suppress the chromosome loss defect of the *ulp2-SIM*^*3A*^*CCR*^*3A*^ mutant. Interestingly, the mutation of *smt3-allR* alone resulted in a drastic 57-fold increase in the rate of chromosome-III loss, which is comparable to that of the *ulp2-SIM*^*3A*^*CCR*^*3A*^ mutant. This suggests that poly-sumoylation is needed to prevent chromosome loss in wild-type cells. Remarkably, combining the *smt3-allR* and *ulp2-SIM*^*3A*^*CCR*^*3A*^ mutations led to a marked reduction in the rate of chromosome loss by ~10-fold (from ~60-fold to 6-fold) (Fig 6A and S8 Table), raising the hypothesis that CCAN sumoylation must be maintained at a precise level to ensure accurate chromosome segregation. To explore this further, we next used the Ulp1-C580S pull-down approach to investigate the effect of *smt3-allR* on the levels of CCAN sumoylation. As shown in Fig 6B, *smt3-allR* has little detectable effect on the amount of sumoylated Mcm21 in the wild-type background, indicating that poly-sumoylated Mcm21 in wild-type cells, if present, exists at a level below the detection limit of this assay. However, *smt3-allR* appears to reduce the amount of the slowest migrating species of Mcm21 in the *ulp2-SIM*^*3A*^*CCR*^*3A*^ mutant (Fig 6B), suggesting that these are the poly-sumoylated species of Mcm21. However, multiple higher molecular weight species of Mcm21 still persist in the *smt3-allR ulp2-SIM*^*3A*^*CCR*^*3A*^ triple mutant (Fig 6B). Because *smt3-allR* is expected to eliminate all branched poly-SUMO chains, these higher molecular weight species of Mcm21 are likely attributed to mono-sumoylation of multiple lysines on Mcm21, although we cannot exclude the possibility that Smt3-allR can still form linear chains whose existence have yet to be confirmed. Similarly, *smt3-allR* does not have an appreciable effect on the abundance of sumoylated Ame1, Okp1, Mcm16 and Mcm22 in the wild-type background (Fig 6C–6F), indicating that poly-sumoylated species of these proteins are below the detection limit of this assay. As observed above and here (Figs 2 and 6C–6F), multiple slower migrating sumoylated species of these CCAN subunits are readily detected in the *ulp2-SIM*^*3A*^*CCR*^*3A*^ mutant, which remain in the *smt3-allR ulp2-SIM*^*3A*^*CCR*^*3A*^ triple mutant, suggesting that each of these CCAN subunits is likely mono-sumoylated on several lysine residues. Interestingly, multiple mono-sumoylated species of Mcm16, Mcm22, Okp1 and Mcm21 appear to accumulate to a higher level in the *smt3-allR ulp2-SIM*^*3A*^*CCR*^*3A*^ triple mutant than in the *ulp2-SIM*^*3A*^*CCR*^*3A*^ mutant. One possible explanation is that *smt3-allR* could channel the unstable poly-sumoylated proteins into the more stable mono-sumoylated proteins, although alternative explanation could also exist; for example, the gel electrophoretic mobility pattern for poly-sumoylated proteins may differ from that of multiply mono-sumoylated species. Regardless of whether poly-SUMO chains could form, a relatively large number of SUMO moieties remain attached to CCAN in the *ulp2-SIM*^*3A*^*CCR*^*3A*^ mutant, which creates a situation where a high local concentration of SUMO at the inner kinetochore could act as a negative feedback signal to recruit Ulp2. Using this feedback mechanism, Ulp2 prevents excessive sumoylation at the inner kinetochore.

### Regulation of CCAN sumoylation during the cell cycle and in response to DNA replication stress

**Fig 7:**
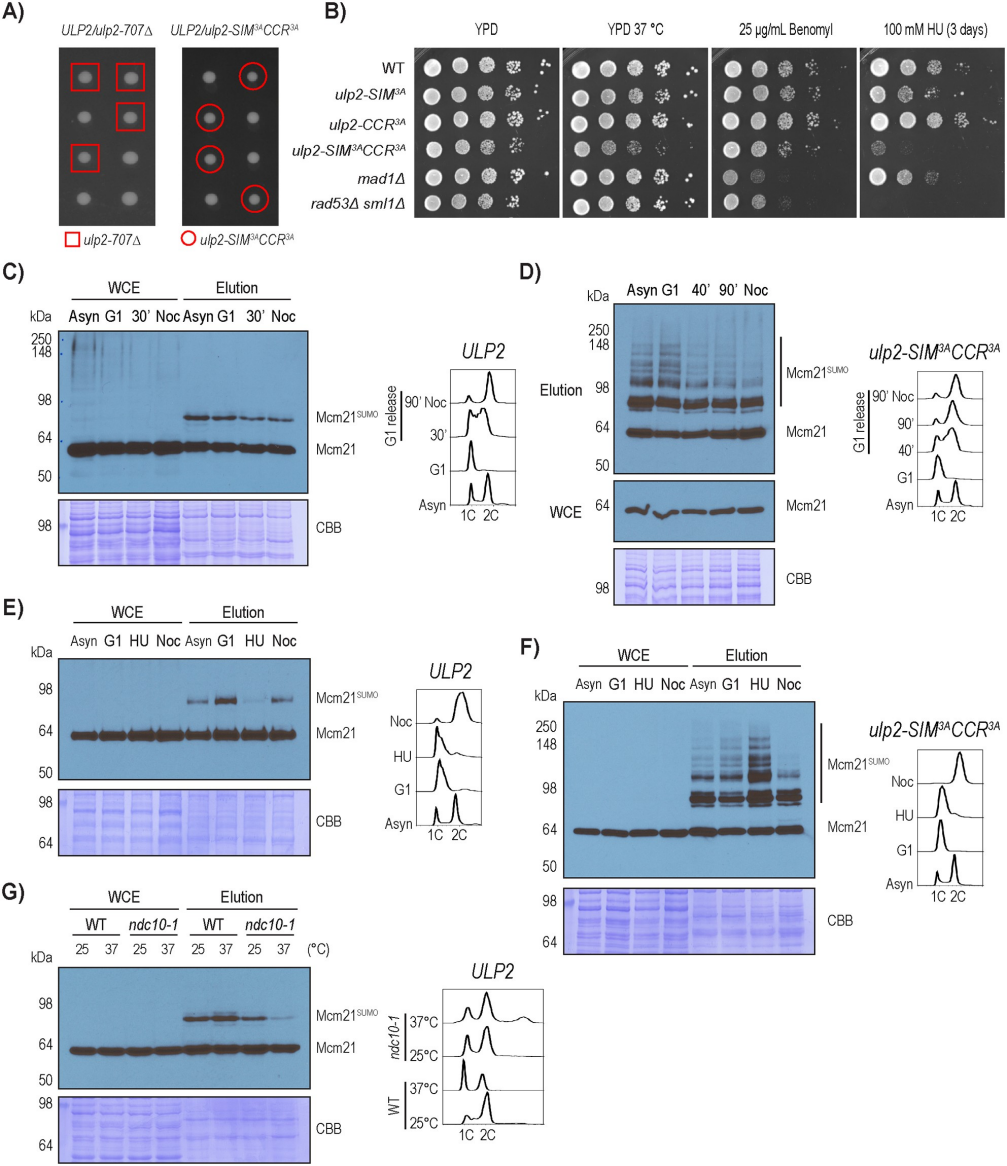
Regulation of Mcm21 desumoylation during the cell cycle and in response to DNA replication stress. A) Tetrad dissection of *ulp2-707* and *ulp2-SIM*^*3A*^*CCR*^*3A*^. B) Plating assay to evaluate the growth of various *ulp2* mutants in response to high temperature, HU and benomyl. As controls, the *rad53Δ* mutant shows an expected hypersensitivity to HU, while the *mad1Δ* mutant shows an expected hypersensitivity to benomyl. C-D) Sumoylation of Mcm21 in wild-type and the *ulp2-SIM*^*3A*^*CCR*^*3A*^ mutant strains during different stages of the cell cycle, which are confirmed by FACS analysis. E-F) Effect of HU treatment on sumoylated Mcm21 in wild-type and the *ulp2-SIM*^*3A*^*CCR*^*3A*^ mutant. G) Effect of *ndc10-1* on sumoylated Mcm21 in the wild-type strain background. Both wild type and *ndc10-1* mutant cells were grown at room temperature, shifted to 37 °C for 6 hours, and then the cells were collected for FACS and Ulp1-C580S pull-down experiments.

To further explore the functions of Ulp2, we performed tetrad dissections of diploid yeast containing *ULP2/ulp2-707Δ* and *ULP2/ulp2-SIM*^*3A*^*CCR*^*3A*^ heterozygous mutants. As seen in Fig 7A, neither of these *ulp2* mutants has an appreciable effect on spore viability or cell growth, unlike the *ulp2Δ* mutant [23]. To determine whether the *ulp2-SIM*^*3A*^*CCR*^*3A*^ double mutant has aneuploidy similar to that of the *ulp2Δ* mutant [27, 39], we applied TMT-based quantitative MS to measure the abundance of proteins expressed from each chromosome. As summarized in S2 Fig and S10 and S11 Tables, chromosomal protein expression levels were not significantly altered between the wild-type and *ulp2-SIM*^*3A*^*CCR*^*3A*^ haploid spores, while elevated levels of protein expression are seen from chromosome I, III and XI in the *ulp2Δ* cells. However, the expected two-fold increase was not observed from any of these chromosomes, likely due to a heterogeneous distribution of aneuploidy in the *ulp2Δ* mutant, which rapidly develops survivors and was found to be genetically unstable, undergoing adaptive evolution and losing its state of aneuploidy [39].

The observation that the *ulp2-SIM*^*3A*^*CCR*^*3A*^ mutant has relatively normal growth comparable to the wild-type strain prompted us to investigate its growth under stresses. Interestingly, these *ulp2* mutants are hypersensitive to hydroxyurea (HU) but not to benomyl (Fig 7B), suggesting that the accumulation of hyper-sumoylated CCAN in the *ulp2* mutant does not impair kinetochore function during chromosome segregation; instead, it may interfere with the kinetochore in response to DNA replication stress, possibly when the centromeres are replicated. Next, we examined the level of sumoylated Mcm21 during different cell cycle stages. Sumoylated Mcm21 appears to be modestly higher in G1, lower in the S and G2-M phases in both WT and the *ulp2-SIM*^*3A*^*CCR*^*3A*^ mutant (Fig 7C and 7D). Interestingly, HU treatment caused a reduction in sumoylated Mcm21 in WT cells, but a marked accumulation of hyper-sumoylated Mcm21 in the *ulp2-SIM*^*3A*^*CCR*^*3A*^ mutant (Fig 7E and 7F). The reason for these distinct responses to HU treatment is presently unknown; although they indicate that sumoylation of Mcm21 is particularly sensitive to DNA replication stress. A remaining question is the role of kinetochore assembly on CCAN sumoylation. To address this, we used the *ndc10-1* mutant, which was shown to disrupt kinetochore assembly at non-permissive temperature [40]. Sumoylated Mcm21 is drastically reduced in the *ndc10-1* mutant following a temperature shift to 37 °C for 6 hours (Fig 7E); and FACS analysis shows that the *ndc10-1* mutant accumulates over 2C DNA content, likely as a result of chromosome mis-segregation. This finding, together with the observation that Mcm16 also contributes to sumoylation of Mcm21 (Fig 5C), suggests that sumoylation of Mcm21 likely occurs on the fully assembled kinetochore at the centromere where Ulp2 is recruited (see Fig 4E).

## Discussion

**Fig 8:**
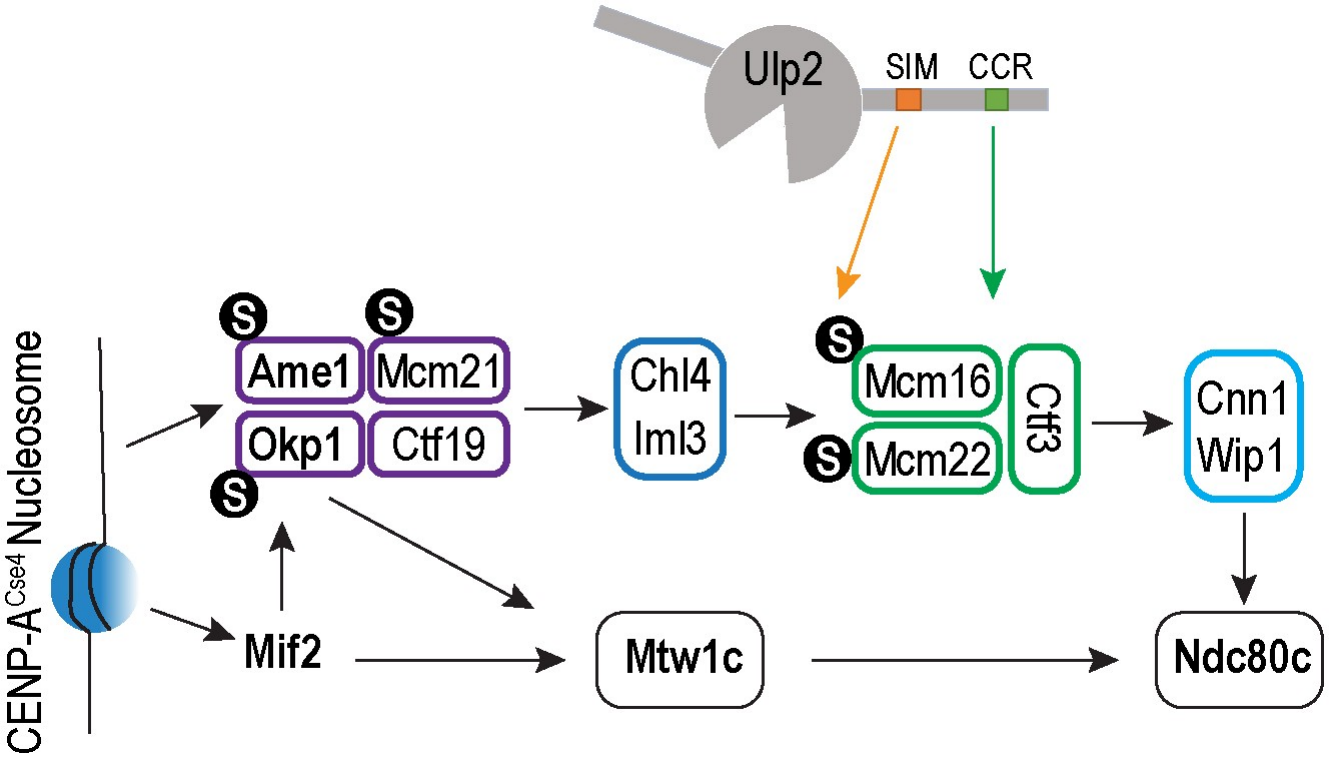
A model of how the Ulp2 protease is recruited to the kinetochore, via binding to the CMM complex and SUMO, to desumoylate the CCAN complex.

Prior studies have implicated that protein sumoylation plays an evolutionarily conserved, yet poorly understood role in regulating kinetochore function. Among the enzymes that catalyze reversible sumoylation, the Ulp2^SENP6^ family of enzymes appears to play a specific role in regulating chromosome segregation [23, 25, 28, 29, 41, 42]; however, the mechanism has been elusive. Our results here show that Ulp2 targets the inner kinetochore CCAN complex through two distinct and partially redundant mechanisms (Fig 8): one that utilizes a newly identified kinetochore-targeting motif (previously referred to as CCR) of Ulp2, which recruits it to the kinetochore via the CMM complex, and the second that applies a negative feedback mechanism to selectively target hyper-sumoylated CCAN subunits via the SIM motif of Ulp2. Importantly, a failure in either mechanism results in an elevated chromosome loss rate, while a loss of both leads to a synergistic increase in chromosome loss. Interestingly, the *smt3-allR* mutant, which reduces the level of poly-sumoylation, results in an elevated rate of chromosome loss in cells containing wild-type Ulp2 (Fig 6); however, the same mutation suppresses the chromosome loss defect of the *ulp2* mutant where CCAN becomes aberrantly poly-sumoylated. Moreover, hyper-sumoylated Mcm21 accumulates in this *ulp2* mutant in response to DNA replication stress, resulting in impaired growth (Fig 7). Thus SUMO homeostasis at the kinetochore is critical to ensure accurate chromosome segregation likely via centromere replication.

Our previous study showed that Ulp1 is responsible for the bulk of intracellular desumoylation, while in contrast Ulp2 is highly specific [30]. This finding was followed by two recent studies, which showed that Ulp2 targets its substrates using a dual substrate recognition mechanism [31, 32]. The findings here further extend this, revealing a new negative feedback mechanism by which Ulp2 targets the kinetochore, where it selectively targets hyper-sumoylated CCAN subunits to maintain SUMO homeostasis. Notably, the *smt3-allR* mutation, which eliminates the formation of branched poly-SUMO chains [43], does not appreciably alter the level of sumoylated CCAN subunits in cells containing wild-type Ulp2, unlike the effect of the *ulp2-SIM*^*3A*^ mutation, which eliminates this negative feedback mechanism altogether. Thus, the binding between the SIM and branched poly-SUMO chains alone is insufficient to account for the negative feedback mechanism of Ulp2. Instead, the fact that several CCAN subunits are modified by multiple SUMO moieties in the *ulp2 smt3-allR* mutant (Fig 7) suggests that mono-sumoylation of multiple lysines in the CCAN complex could create a high local concentration of SUMO at the kinetochore, collectively triggering Ulp2’s negative feedback mechanism. Consistent with this idea, sumoylation of CCAN requires a functional Ndc10, suggesting that the assembled kinetochore is being targeted. Considering that SUMO has been found to modify multiple subunits of many protein complexes [33, 44–46], it is conceivable that Ulp2 might employ a similar negative feedback mechanism to target other substrates, particularly when their sumoylation is allowed to accumulate.

The findings here suggest that homeostasis of kinetochore sumoylation, specifically by Ulp2, is critical for accurate chromosome segregation. However, the specific function of sumoylation at the kinetochore or the specific role that sumoylated CCAN plays remain key questions for future investigation. The observation that several CCAN subunits can be sumoylated at multiple lysine residues or via poly-SUMO chains raises the possibility that these sumoylation events may act collectively to regulate the kinetochore, aside from acting as Ulp2’s negative feedback signal. Concerning the nature of this function, a previous study showed that the knockdown of SENP6 in human cells led to the mis-localization of the CENP-H/I/K complex [29], the human ortholog of *S*. *cerevisiae* CMM. However, deletion of Mcm16, a core subunit of the CMM complex, does not fully recapitulate the chromosome segregation defect of the *ulp2* mutant (Fig 5A), suggesting that the role that Ulp2 plays in regulating the kinetochore is not limited to maintaining the function of CMM in yeast. Instead, the fact that *mcm16Δ* strongly suppresses the chromosome loss defect of the *ulp2* mutant appears to be consistent with the requirement for Mcm16 in generating an aberrantly hyper-sumoylated kinetochore in the *ulp2* mutant. On the other hand, the reduction of poly-sumoylation in the *smt3-allR* mutant results in a substantial increase in chromosome loss rate (Fig 6), indicating that poly-sumoylation plays an important role in maintaining accurate chromosome segregation in wild-type cells. Although the *smt3-allR* mutation does not appreciably affect the amount of sumoylated CCAN subunits in unperturbed wild-type cells (Fig 6), we cannot exclude the possibility that poly-sumoylated CCAN may accumulate transiently and is thus too low to be detected in unperturbed cells. Strikingly, the combination of the *smt3-allR* and *ulp2-SIM*^*3A*^*CCR*^*3A*^ mutations led to a mutual suppression of the chromosome loss defect seen in the individual mutants (Fig 6A), further supporting the idea that the level of sumoylation at the CCAN complex must be tightly regulated to ensure accurate chromosome segregation.

What might the function of sumoylated CCAN be? Given the relatively low stoichiometry of sumoylated CCAN subunits, it seems appropriate to consider them an unstable intermediate, which trigger a biological response only when needed, with Ulp2 preventing their accumulation under normal circumstances. Interestingly, the *ulp2* mutants are hypersensitive to DNA replication stress, but not to benomyl that specifically perturbs chromosome segregation (Fig 7B). Moreover, DNA replication stress strongly perturbs the amount of sumoylated CCAN (Fig 7E and 7F). Although still speculative, these findings suggest that sumoylated CCAN may act during centromere replication when the kinetochore is expected to undergo a dynamic remodeling. If so, how the kinetochore-centromere interface is perturbed in the *ulp2* mutant remains an important subject for future investigations.

## Materials and methods

### *S*. *cerevisiae* strain and plasmid construction

Standard *S*. *cerevisiae* genetic methods were used to generate strains and plasmids in this study (S12 Table). All integrated mutations in yeast strains and plasmids were confirmed by PCR and DNA sequencing.

### Yeast growth assay

Yeast growth was measured using a 5-Fluoroorotic acid (5-FOA) sensitivity assay as previously described [31]: Briefly, cells were grown in 4 mL of synthetic complete medium lacking leucine (SC-Leu, US Biological) until OD_600_ ~1, followed by normalization to an OD_600_ of 0.5. Cells were then diluted five-fold serial dilutions in a sterile 96-well plate with sterile dH_2_O. 5 μL of each dilution was then spotted on either SC-Leu plates or 5-FOA plates (SC supplemented with 0.1% 5-FOA). Both plates were incubated at 30°C for two days, and images were acquired using a Bio-Rad ChemiDoc MP imaging system. Similarly, wild-type and various mutant cells were grown in YPD, 10-fold serial diluted and then spotted on plates containing either hydroxyurea or benomyl (see Fig 7).

### Chromosome loss assay

Chromosome loss rates were measured using a quantitative mating assay as previously described [37]. Briefly, *ulp2* mutants, expressed in haploid *MATα ARG2 LEU2 ura3* strains, were mated to a tester strain (HZY601: *MATα arg2 URA3*) by mixing ~1×10^7^ log-phase cells of each strain on a filter membrane (0.8 μm MCE Membrane Filter, MF-Millipore), followed by incubation at 30°C for 5 h on a YPD plate (1% Yeast extract, 2% Peptone and 2% Dextrose). Cells were washed and resuspended in 1 mL of sterile dH_2_O. The total number of viable cells was determined by taking 1% of the cell population and plating them onto SC-Arg plates. Diploid cells were selected by plating 10–100% of the remaining cells onto SC-Arg-Ura plates, such that the final numbers of cells growing on SC-Arg-Ura plates were between 100 and 200. Chromosome loss rates were determined by taking the number of Arg^+^ Ura^+^ Leu^−^colonies divided by the total number of viable colonies. For each experimental strain, 95% confidence intervals, of the median chromosome loss rates, were calculated using 16 isolates from each strain.

### Protein purification and preparation of CNBr-activated resin

Ulp1^403-621^-C580S affinity resin was generated by cloning Ulp1^403-621^-C580S into a LIC 2C-T plasmid containing an N-terminal, TEV-cleavable, 6×His-MBP fusion tag. This construct was transformed into *Escherichia coli* Rosetta-2(DE3) pLysS (Novagen) cells, and grown in 2 liters of LB (Luria Broth) media containing 100 μg/mL of ampicillin and 34 μg/mL of chloramphenicol. Proteins were expressed using IPTG induction (0.2 mM IPTG) for 16h at 18°C, with cells being induced at a starting OD_600_ of ~0.6. Cells were lysed in PBSN buffer (1.06 mM KH_2_PO_4_, 5.6 mM K_2_HPO_4_, 154 mM NaCl, 10% Glycerol, 0.2% NP-40, pH 7.4) with protease inhibitors (2 mM phenylmethylsulfonyl fluoride, 200 μM benzamidine, 0.5 μg/mL leupeptin, 1 μg/mL pepstatin A). Ulp1^403-621^-C580S was purified via Ni-NTA resin (Qiagen), followed by dialysis and anion exchange chromatography (monoQ 5/50 GL) on an ÄKTA pure FPLC system. Elution fractions were pooled and concentrated to a final protein concentration of 15 mg/mL; proteins were then conjugated to CNBr-activated resin (GE Healthcare) according to manufacturer protocols. Ulp2-CCR resin was prepared in the same manner as Ulp1^403-621^-C580S resin with the following modification: Ulp2^873-1034^ WT or mutant variants were cloned into a LIC 2G-T plasmid containing an N-terminal, TEV-cleavable, 6×His -GST fusion tag.

### Analysis of CCAN sumoylation and protein-protein interactions

To analyze sumoylated CCAN proteins, whole cell extracts were generated by glass bead beating, followed by the enrichment of sumoylated proteins via Ulp1-C580S affinity resin. Specifically, yeast cells were grown in 100 mL of YPD or SC-Leu medium to an OD_600_ of 1.0. Cells were harvested and washed with 10 mL of PBS buffer (PBS, 10% Glycerol, pH 7.4), supplemented with protease inhibitors (2 mM phenylmethylsulfonyl fluoride, 200 μM benzamidine, 0.5 μg/mL leupeptin, 1 μg/mL pepstatin A), 20 mM N-Ethylmaleimide and 20 mM Iodoacetamide. Cell pellets were resuspended in 1 mL of PBSN buffer, containing 2 mM MgCl_2_, protease inhibitors and 100 μg DNaseI (Grade II, Roche). Whole cell extracts were generated at 4°C via glass bead-beating (500 μL of glass beads, 10 cycles of 30s break with 2 min rest period) and followed by centrifugation at 15,000 ×*g*. Sumoylated proteins were purified by incubating soluble whole cell extracts with 20 μL Ulp1^403-621^-C580S resin for 2h at 4°C. Resin was washed six times with PBSN buffer, and bound proteins were eluted by boiling in 25 μL of 2×LDS sample buffer (NuPAGE LDS Sample Buffer, Invitrogen). Samples were then reduced using 50 mM DTT and run on SDS-PAGE and analyzed by western blotting using an α-Protein A 1° antibody (Sigma P3775), α-Smt3 1° antibody (rabbit polyclonal antibody made via Covance, Inc.), and an α-rabbit HRP 2° antibody (Sigma). To test if the slower migrating species of Ame1 are sumoylated (S1 Fig), cells expressing Protein A tagged Ame1 were harvested and lysed as above with the following modifications: Omission of N-ethylmaleimide and Iodoacetamide from the cell washing buffer, and the addition of 2 mM DTT and 2.5 μg of recombinant Ulp1 to ensure that SUMO conjugates have been cleaved off of target proteins [35].

To analyze the interactions between CCAN subunits and Ulp2-CCR, pull-down assays were performed using either wild type or mutant variants of Ulp2-CCR resin. Pulldown assays were performed in the same manner as described above for the enrichment of sumoylated proteins using Ulp1^403-621^-C580S. To detect binding between Ctf3 and CCR peptides, biotinylated peptides of UIp2^896-937^-CCR and UIp2^896-937^-CCR^3A^ (0.2 mM from EZbio) in 1 mL PBS were preincubated with 100 μL of NeutrAvidin-agarose resin and washed with 1 mL of PBSN. Whole cell extracts containing Ctf3-Protein A were incubated with 10 μL of peptide resin and washed with 5×1 mL of PBSN buffer. Bound proteins were eluted using 25 μL of 2×LDS sample buffer and then run on SDS-PAGE followed by Protein a western blot as described above.

### ChIP analysis of Ulp2

To analyze the localization of Ulp2 to centromere, ChIP was carried out as described previously [47]. Briefly, yeast cultures (50 mL for each immunoprecipitation) were grown to an OD_600_ of 0.8 and cross-linked for 15 min with 1% formaldehyde at room temperature followed by the addition of 125 mM glycine to quench the reaction. Whole cell lysates were prepared via glass bead-beating as described above and sonicated to shear the genomic DNA to an average size of 500 bp. Immunoprecipitation was performed using Dynabeads Protein G and anti-Flag antibody M2 (5 μL for each IP, Sigma) and then the Input and IP material were purified using QIAquick PCR Purification kit (QIAGEN). The Input was diluted 1:100 and IP samples were diluted 1:10 in water followed by qPCR using SYBR Green 2x master mix (KAPA Biosystems) on a Roche LightCycler 480. Genomic DNA prepared from wild-type cells was serially diluted to make a standard curve of each primer pair for calculation. Fold enrichment values were calculated as percentage of total Input DNA. Primer pairs used here: CEN3-Forward ATCAGCGCCAAACAATATGGAAAA, CEN3-Reverse GAGCAAAACTTCCACCAGTAAACG, CUP1-Forward AACTTCCAAAATGAAGGTCA and CUP1-Reverse GCATGACTTCTTGGTTTCTT.

### Cell cycle arrest and FACS analysis

To analyze sumoylation of Mcm21 during cell cycle (Fig 7), wild-type and *ulp2-SIM*^*3A*^*CCR*^*3A*^ mutant (containing *bar1Δ*) were arrested in the G1 phase by 15 nM alpha-factor for 3.5 hours. Cells were then washed twice with pre-warmed fresh YPD media and then suspended in two volumes of pre-warmed fresh YPD media to allow re-entry into the S phase. At each time point, aliquots of cells were harvested for Ulp1^403-621^-C580S pull-down analysis as described above. In parallel, 300 μL cell culture of each time point was fixed by 700 μL ethanol, treated by Protease K and RNAse A, and then stained by Sytox Green dye for FACS analysis using a BD LSRFortessa cell analyzer. 200 mM HU or 7.5 μg/mL nocodazole was added to various yeast cultures for 3 hours to arrest cells in the early S or G2-M phase, respectively. These arrested cells were then similarly processed for WB and FACS analyses.

### Preparation of sumoylated proteins from yeast whole cell extracts and TMT labeling of peptides for LC-MS/MS analysis

Total sumoylated proteins from yeast strains containing HF-Smt3 were purified as previously described [31]. To generate yeast whole cell extracts for Tandem Mass Tag (TMT) labeling, yeast cells were grown up in YPD medium at 30°C to an OD_600_ of 1.0. Cells were pelleted by centrifugation at 4000 ×*g* at 4°C, followed by glass bead beating for 10 min in lysis buffer (200 mM NaOH, 100 mM Phosphate Buffer pH 8.0, 2% SDS). Samples were neutralized using 200 mM HCl and boiled at 65°C for 10 min. Protein concentrations were then measured by Bradford reagent and equal amounts of protein (~200 μg) from each extract were digested using 2 μg of trypsin protease at 37°C. Protein digests were acidified using Trifluoroacetic acid (TFA) to a final concentration of 0.5%. Digested peptides were desalted using C18 Sep-Pak cartridges (Waters) and dried down by speed-vac at 30°C. Thermo Scientific TMT10plex labels were used to label tryptic peptides generated from yeast whole cell extracts. Specifically, TMT10-126, TMT10-127N, TMT10-128C, TMT10-129N, TMT10-130C were used to differentially label samples. Briefly, 20 μg of peptides from yeast whole cell extract were labeled with an individual TMT10 tag for 2h at room temperature. Samples were then combined and dried down by speed-vac at 30°C. Peptides were then re-suspended in 80% acetonitrile 20% dH2O and subjected to offline fractionation as previously described [33]. Fig 5C used TMT10-127N, TMT10-128C, TMT10-129N and TMT10-130C. Fig 5D used TMT10-126, TMT10-127N, TMT10-128C, TMT10-129N and TMT10-130C.

### Sf9 insect cell expression of Ctf3-Mcm16-Mcm22 and pulldown assay with Ulp2-CCR resin

SF9 insect cell co-expression of Ctf3, Mcm16 and Mcm22 was performed as described in the Baculovirus Expression System (Thermo Fisher Scientific). The co-expression construct was derived from yeast genomic DNA and sequentially cloned and inserted into the pFastbac plasmid using standard restriction enzyme and DNA ligation. Whole cell extracts were generated from 200 mL of SF9 insect cells at ~ 2.0 x 10^6^ cells/mL. Cells were harvested by centrifugation at 400 *×g* at 4°C, followed by washing with 10 mL of wash buffer (PBS, 10% glycerol, 2 mM phenylmethylsulfonyl fluoride, 200 μM benzamidine, 0.5 μg/mL leupeptin, 1 μg/mL pepstatin A and 100 μg/mL of DNaseI). Cell pelleted were dounced 20 times with a 15 mL dounce homogenizer and spun down at 30,000 *×g* at 4°C. 1 mL of clarified cell extracts were then bound to 25 μL Ulp2-CCR or Ulp2-CCR^3A^ resin for 2 hours at 4°C. Resin was washed with 8 volumes of PBS + 0.2% NP-40 and eluted using 150uL of elution buffer (6M Urea + 50mM phosphate for 1 hour at 4°C. Elutions were reduced, alkylated and digested with Trypsin (1 μg per elution) as described above. Samples were then acidified and labeled using TMT labels as described above.

### Data analysis for LC-MS/MS data

Data analysis for SILAC labeled samples were performed as previously described [31] with the following exception: A minimum cut-off for 3 unique peptides was not applied. Instead proteins with less than 3 unique peptides were validated by manual inspection. TMT labeled samples were searched via the COMET peptide search engine as part of the Trans Proteomic Pipeline (TPP). The search results were then processed using TPP, where quantification of the TMT reporter ion was analyzed by the Libra software tool. An FDR of less than 1% was applied for peptide identification, and a minimum intensity of the TMT reporter ion was set at 1000. Expression of chromosome specific proteins were quantified by averaging the contributing signal of each TMT reporter ion that originates from proteins belonging to each of the 16 yeast chromosomes.

The MS data analysis of SF9 expressed CMM extracts was performed in the same manner as above, with the following modifications: a composite protein database, containing the SF9 insect cell proteome along with the yeast protein sequence of Ulp2, Ctf3, Mcm16 and Mcm22 was used to search the MS data; additionally, a 3 peptide minimum cut-off was applied.

## Supporting information

S1 Fig A) Western blot of Protein A tagged Ame1 from whole cell extracts and enriched sumoylated proteins. The upper band of Ame1 in whole cell extract (WCE), as indicated by an asterisk, was insensitive to Ulp1 treatment. In contrast, the eluted sample contains an upper band, which is sensitive to Ulp1 treatment and is thus the sumoylated species of Ame1. B) Conjugation of purified Ulp2-CCR (WT and 3A mutant) proteins to CNBr resin. These resins were then used to examine the binding between the Ulp2-CCR and CCAN subunits (see Fig 3).
(DOCX)

S2 Fig Quantitative MS to compare chromosome-specific protein expression between wild type and various *ulp2* mutants. Expression of chromosome specific proteins were quantified by averaging the contributing signal of each TMT reporter ion that originates from proteins encoded by genes on each of the 16 yeast chromosomes. A) Comparison of chromosome-specific expression of proteins between WT and *ulp2-SIM*^*3A*^*CCR*^*3A*^ mutant. B) Comparison of chromosome-specific expression of proteins between WT and four independent *ulp2Δ* strains.
(DOCX)

S1 Table Quantitative MS to compare sumoylated proteins in WT and the *ulp2-CCR*^*3A*^ mutant. Median ratios and the number of positive spectral matches (PSMs) are listed for each protein.
(DOCX)

S2 Table Quantitative MS to compare sumoylated proteins in the *ulp2-SIM*^*3A*^ and the *ulp2-SIM*^*3A*^*CCR*^*3A*^ mutants. Median ratios and the number of positive spectral matches (PSMs) are listed for each protein.
(DOCX)

S3 Table Quantitative MS to compare sumoylated proteins in WT and the *ulp2-SIM*^*3A*^ mutant. Median ratios and the number of positive spectral matches (PSMs) are listed for each protein.
(DOCX)

S4 Table Quantitative MS to compare sumoylated proteins in the *ulp2-CCR*^*3A*^ and the *ulp2-SIM*^*3A*^*CCR*^*3A*^ mutants. Median ratios and the number of positive spectral matches (PSMs) are listed for each protein.
(DOCX)

S5 Table Quantitative MS to compare sumoylated proteins in WT and the *ulp2-SIM*^*3A*^*CCR*^*3A*^ mutant. Median ratios and the number of positive spectral matches (PSMs) are listed for each protein.
(DOCX)

S6 Table Quantitative MS to compare sumoylated proteins in the *ulp2Δ* and the *ulp2-CCR*^*3A*^*SIM*^*3A*^ mutants. Median ratios and the number of positive spectral matches (PSMs) are listed for each protein.
(DOCX)

S7 Table Quantitative MS to compare the binding proteins of the wild-type Ulp2-CCR and the Ulp2-CCR^3A^ resins, using SF9-insect cell extracts expressing the yeast CMM complex. % abundance, standard-error of the mean (SEM), average abundance ratios and the number of positive spectral matches (PSMs) for proteins associating with CCR and CCR^3A^ resin are listed.
(DOCX)

S8 Table Quantitative mating results for assaying the rate of chromosome III loss, which are used to generate Figs 5B, 6A and 7A. Median chromosome III loss rates, the 95% confidence interval (CI) and the Fold-change relative to WT are shown.
(DOCX)

S9 Table Quantitative MS to compare sumoylated proteins in the *ulp2Δ* and *ulp2Δmcm16Δ mutants*. Median ratios and the number of positive spectral matches (PSMs) are listed for each protein.
(DOCX)

S10 Table Quantitative MS to compare the % abundance of proteins expressed on each chromosome in wild-type and the *ulp2-SIM*^*3A*^*CCR*^*3A*^ mutants. (DOCX)

S11 Table Quantitative MS to compare the % abundance of proteins expressed on each chromosome in wild-type and several independently prepared *ulp2Δ* mutants. (DOCX)

S12 Table Yeast strains and plasmids used in this study. (DOCX)
