# Supplementary material for: Recruitment of the Ulp2 protease to the inner kinetochore prevents its hyper-sumoylation to ensure accurate chromosome segregation
Source: PLoS Genet. 2019 Nov 20;15(11):e1008477. doi: 10.1371/journal.pgen.1008477 (PMC6892545; doi:10.1371/journal.pgen.1008477)

**S1 Figure**. A) Western blot of Protein A tagged Ame1 from whole cell extracts and enriched sumoylated proteins. The upper band of Ame1 in whole cell extract (WCE), as indicated by an asterisk, was insensitive to Ulp1 treatment. In contrast, the eluted sample contains an upper band, which is sensitive to Ulp1 treatment and is thus the sumoylated species of Ame1. B) Conjugation of purified Ulp2-CCR (WT and 3A mutant) proteins to CNBr resin. These resins were then used to examine the binding between the Ulp2-CCR and CCAN subunits (see Figure 3).


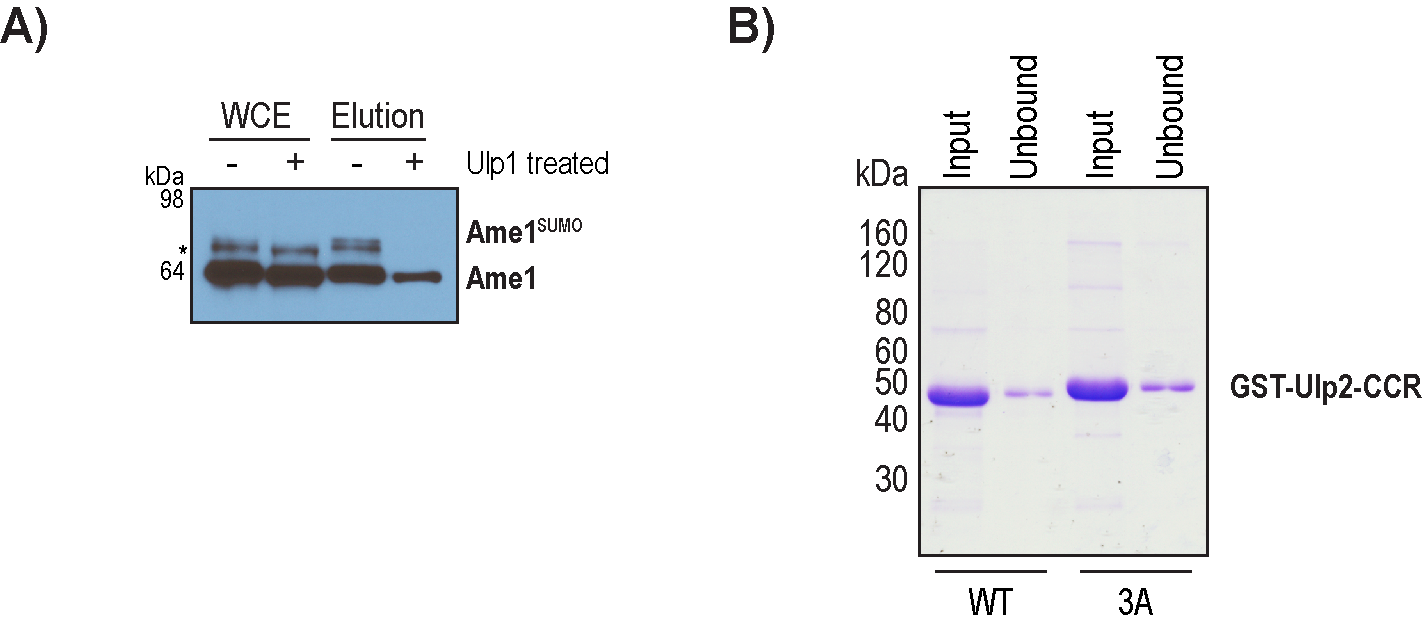

Supplement: S1 Fig — A) Western blot of Protein A tagged Ame1 from whole cell extracts and enriched sumoylated proteins. The upper band of Ame1 in whole cell extract (WCE), as indicated by an asterisk, was insensitive to Ulp1 treatment. In contrast, the eluted sample contains an upper band, which is sensitive to Ulp1 treatment and is thus the sumoylated species of Ame1. B) Conjugation of purified Ulp2-CCR (WT and 3A mutant) proteins to CNBr resin. These resins were then used to examine the binding between the Ulp2-CCR and CCAN subunits (see Fig 3). (DOCX) [file pgen.1008477.s001.docx]
