# Supplementary material for: Recruitment of the Ulp2 protease to the inner kinetochore prevents its hyper-sumoylation to ensure accurate chromosome segregation
Source: PLoS Genet. 2019 Nov 20;15(11):e1008477. doi: 10.1371/journal.pgen.1008477 (PMC6892545; doi:10.1371/journal.pgen.1008477)
Supplement: S2 Fig — Expression of chromosome specific proteins were quantified by averaging the contributing signal of each TMT reporter ion that originates from proteins encoded by genes on each of the 16 yeast chromosomes. A) Comparison of chromosome-specific expression of proteins between WT and ulp2-SIM3ACCR3A mutant. B) Comparison of chromosome-specific expression of proteins between WT and four independent ulp2Δ strains. (DOCX) [file pgen.1008477.s002.docx]

**S2 Figure**. Quantitative MS to compare chromosome-specific protein expression between wild type and various *ulp2* mutants. Expression of chromosome specific proteins were quantified by averaging the contributing signal of each TMT reporter ion that originates from proteins encoded by genes on each of the 16 yeast chromosomes. A) Comparison of chromosome-specific expression of proteins between WT and *ulp2-SIM^3A^CCR^3A^* mutant. B) Comparison of chromosome-specific expression of proteins between WT and four independent *ulp2Δ* strains.

**
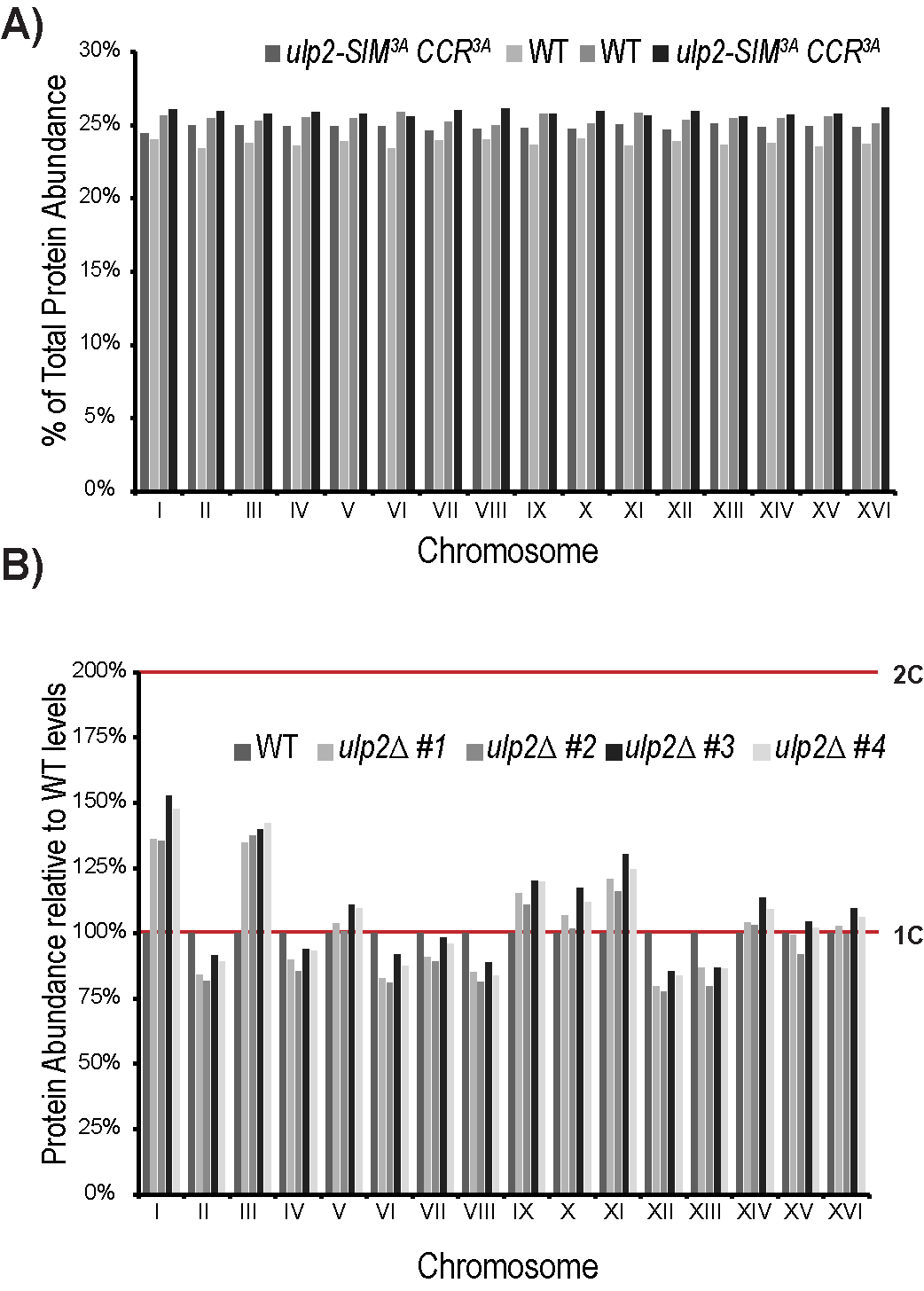
**
