# Supplementary material for: Recruitment of the Ulp2 protease to the inner kinetochore prevents its hyper-sumoylation to ensure accurate chromosome segregation
Source: PLoS Genet. 2019 Nov 20;15(11):e1008477. doi: 10.1371/journal.pgen.1008477 (PMC6892545; doi:10.1371/journal.pgen.1008477)
Supplement: S3 Table — Median ratios and the number of positive spectral matches (PSMs) are listed for each protein. (DOCX) [file pgen.1008477.s005.docx]

**S3 Table**. Quantitative MS to compare sumoylated proteins in WT and the *ulp2-SIM^3A^* mutant. Median ratios and the number of positive spectral matches (PSMs) are listed for each protein.

| Gene | ORF | Median *SIM^3A^*/WT | # of Peptide IDs |
| --- | --- | --- | --- |
| ABF1 | YKL112W | 0.5 | 39 |
| ABP1 | YCR088W | 0.4 | 10 |
| AME1 | YBR211C | 2.5 | 2 |
| AOS1 | YCR088W | 0.4 | 10 |
| ASF2 | YDL197C | 0.7 | 24 |
| AZF1 | YOR113W | 0.4 | 17 |
| BDF1 | YLR399C | 0.8 | 28 |
| BDP1 | YNL039W | 2.8 | 53 |
| BIR1 | YJR089W | 1.0 | 92 |
| BOP3 | YNL042W | 0.3 | 23 |
| BRF1 | YGR246C | 1.8 | 25 |
| BRN1 | YBL097W | 1.4 | 38 |
| BUD3 | YCL014W | 1.0 | 18 |
| BUD4 | YJR092W | 1.0 | 24 |
| CBF1 | YJR060W | 0.7 | 10 |
| CBF2 | YGR140W | 1.7 | 43 |
| CDC11 | YJR076C | 0.9 | 10 |
| CDC14 | YFR028C | 9.4 | 42 |
| CDC3 | YLR314C | 1.1 | 32 |
| CDC46 | YLR274W | 0.9 | 18 |
| CDC47 | YBR202W | 1.0 | 4 |
| CDC48 | YDL126C | 6.0 | 59 |
| CET1 | YPL228W | 1.0 | 41 |
| CIN5 | YOR028C | 1.6 | 12 |
| CRZ1 | YNL027W | 0.6 | 14 |
| CST6 | YIL036W | 1.0 | 9 |
| CTI6 | YPL181W | 0.5 | 17 |
| CYC8 | YBR112C | 0.6 | 25 |
| DIG1 | YPL049C | 0.5 | 13 |
| EDE1 | YBL047C | 0.5 | 17 |
| ENO2 | YGR254W | 0.9 | 17 |
| FOB1 | YDR110W | 1.4 | 23 |
| GCN5 | YGR252W | 0.6 | 22 |
| GCR1 | YPL075W | 0.5 | 23 |
| GCR2 | YNL199C | 0.6 | 5 |
| GIN4 | YDR507C | 0.8 | 8 |
| HAP1 | YLR256W | 2.0 | 50 |
| HIR2 | YOR038C | 0.4 | 45 |
| HMO1 | YDR174W | 0.5 | 17 |
| HMS1 | YOR032C | 0.4 | 4 |
| HPC2 | YBR215W | 0.4 | 54 |
| HRP1 | YOL123W | 0.5 | 3 |
| HSP104 | YLL026W | 0.9 | 9 |
| HTA1 | YDR225W | 2.2 | 4 |
| HTA2 | YBL003C | 2.2 | 4 |
| HTB1 | YDR224C | 1.8 | 16 |
| HTB2 | YBL002W | 1.8 | 15 |
| IPP1 | YBR011C | 0.9 | 4 |
| IRR1 | YIL026C | 1.6 | 24 |
| ISW1 | YBR245C | 0.6 | 75 |
| ISW2 | YOR304W | 0.5 | 13 |
| ITC1 | YGL133W | 0.4 | 14 |
| KRE33 | YNL132W | 0.4 | 32 |
| MAD1 | YGL086W | 0.5 | 26 |
| MCD1 | YDL003W | 2.9 | 16 |
| MCM16 | YPR046W | 1.7 | 1 |
| MCM2 | YBL023C | 1.1 | 28 |
| MCM21 | YDR318W | 1.9 | 6 |
| MCM3 | YEL032W | 1.8 | 34 |
| MCM6 | YGL201C | 0.5 | 15 |
| MET4 | YNL103W | 0.7 | 25 |
| MLP1 | YKR095W | 0.6 | 184 |
| MLP2 | YIL149C | 0.7 | 147 |
| MOT1 | YPL082C | 0.9 | 38 |
| MRP8 | YKL142W | 0.5 | 19 |
| NBA1 | YOL070C | 0.5 | 8 |
| NET1 | YJL076W | 6.8 | 286 |
| NGG1 | YDR176W | 0.7 | 22 |
| NPL6 | YMR091C | 0.7 | 18 |
| NUP2 | YLR335W | 0.4 | 45 |
| NUP60 | YAR002W | 0.4 | 13 |
| NUT1 | YGL151W | 0.6 | 39 |
| OKP1 | YGR179C | 7.4 | 2 |
| OLA1 | YBR025C | 0.6 | 4 |
| ORC3 | YLL004W | 0.5 | 24 |
| PAA1 | YDR071C | 0.7 | 4 |
| PAF1 | YBR279W | 0.5 | 19 |
| PDC1 | YLR044C | 0.8 | 17 |
| PGK1 | YCR012W | 0.6 | 31 |
| POB3 | YML069W | 0.7 | 20 |
| POL12 | YBL035C | 1.0 | 5 |
| POL30 | YBR088C | 1.3 | 9 |
| PRP45 | YAL032C | 0.5 | 20 |
| RAD16 | YBR114W | 0.5 | 33 |
| RAP1 | YNL216W | 0.6 | 48 |
| REB1 | YBR049C | 0.8 | 55 |
| RET1 | YOR207C | 1.4 | 28 |
| RIF1 | YBR275C | 0.7 | 73 |
| RIS1 | YOR191W | 2.2 | 39 |
| RPA135 | YPR010C | 0.7 | 18 |
| RPA190 | YOR341W | 1.1 | 55 |
| RPB4 | YJL140W | 0.8 | 11 |
| RPC37 | YKR025W | 1.3 | 15 |
| RPC53 | YDL150W | 1.1 | 35 |
| RPC82 | YPR190C | 1.7 | 46 |
| RPO21 | YDL140C | 0.7 | 72 |
| RPO26 | YPR187W | 0.9 | 3 |
| RRP5 | YMR229C | 0.6 | 69 |
| RSC1 | YGR056W | 0.6 | 34 |
| RSC2 | YLR357W | 0.6 | 65 |
| RSC58 | YLR033W | 0.6 | 26 |
| RSC6 | YCR052W | 0.7 | 13 |
| RSC8 | YFR037C | 0.7 | 38 |
| RTT102 | YGR275W | 0.7 | 4 |
| RVB1 | YDR190C | 0.7 | 14 |
| RVB2 | YPL235W | 0.6 | 9 |
| SAW1 | YAL027W | 1.0 | 6 |
| SCS2 | YER120W | 0.9 | 9 |
| SEF1 | YBL066C | 0.5 | 25 |
| SGF73 | YGL066W | 0.6 | 11 |
| SHS1 | YDL225W | 1.0 | 25 |
| SIN3 | YOL004W | 0.6 | 58 |
| SIR3 | YLR442C | 0.6 | 62 |
| SIR4 | YDR227W | 0.7 | 125 |
| SIZ1 | YDR409W | 0.4 | 38 |
| SIZ2 | YOR156C | 1.0 | 22 |
| SKO1 | YNL167C | 0.4 | 33 |
| SLI15 | YBR156C | 1.2 | 37 |
| SMC1 | YFL008W | 1.2 | 50 |
| SMC2 | YFR031C | 1.3 | 66 |
| SMC3 | YJL074C | 1.5 | 62 |
| SMC4 | YLR086W | 1.2 | 99 |
| SMC5 | YOL034W | 0.4 | 63 |
| SMC6 | YLR383W | 0.4 | 52 |
| SNF2 | YOR290C | 0.7 | 77 |
| SPA2 | YLL021W | 0.5 | 29 |
| SPC24 | YMR117C | 0.9 | 10 |
| SPN1 | YPR133C | 0.7 | 15 |
| SPP41 | YDR464W | 0.9 | 104 |
| SPT15 | YER148W | 2.8 | 11 |
| SPT5 | YML010W | 0.7 | 64 |
| SPT7 | YBR081C | 0.5 | 79 |
| STB3 | YDR169C | 0.4 | 24 |
| STE12 | YHR084W | 0.6 | 20 |
| STH1 | YIL126W | 0.6 | 68 |
| STU1 | YBL034C | 0.6 | 46 |
| SUB2 | YDL084W | 0.7 | 12 |
| SUM1 | YDR310C | 0.5 | 102 |
| SWC3 | YAL011W | 0.6 | 34 |
| SWI3 | YJL176C | 0.8 | 27 |
| SWI4 | YER111C | 0.5 | 35 |
| SWR1 | YDR334W | 0.7 | 76 |
| TAF12 | YDR145W | 1.0 | 16 |
| TAF2 | YCR042C | 0.8 | 31 |
| TAF3 | YPL011C | 1.0 | 5 |
| TAF5 | YBR198C | 0.8 | 31 |
| TAL1 | YLR354C | 0.9 | 6 |
| TEC1 | YBR083W | 0.5 | 21 |
| TFC3 | YAL001C | 1.2 | 60 |
| TFC4 | YGR047C | 1.6 | 46 |
| TFC6 | YDR362C | 1.2 | 9 |
| TFC7 | YOR110W | 1.7 | 10 |
| TFG1 | YGR186W | 1.0 | 56 |
| TKL1 | YPR074C | 1.0 | 10 |
| TOA1 | YOR194C | 0.9 | 15 |
| TOF2 | YKR010C | 2.8 | 61 |
| TOP1 | YOL006C | 0.8 | 52 |
| TOP2 | YNL088W | 0.8 | 85 |
| TUP1 | YCR084C | 0.5 | 76 |
| TYE7 | YOR344C | 1.9 | 8 |
| UBA2 | YDR390C | 0.8 | 21 |
| UBC9 | YDL064W | 0.4 | 13 |
| UPC2 | YDR213W | 0.4 | 35 |
| VHR1 | YIL056W | 0.5 | 19 |
| VHR2 | YER064C | 0.5 | 11 |
| VID21 | YDR359C | 0.6 | 50 |
| VPS72 | YDR485C | 0.7 | 54 |
| WTM1 | YOR230W | 0.3 | 12 |
| YCG1 | YDR325W | 1.6 | 33 |
| YCS4 | YLR272C | 1.1 | 42 |
| YMR111C | YMR111C | 0.9 | 42 |
| ZEO1 | YOL109W | 0.6 | 10 |
