# Supplementary material for: Recruitment of the Ulp2 protease to the inner kinetochore prevents its hyper-sumoylation to ensure accurate chromosome segregation
Source: PLoS Genet. 2019 Nov 20;15(11):e1008477. doi: 10.1371/journal.pgen.1008477 (PMC6892545; doi:10.1371/journal.pgen.1008477)
Supplement: S7 Table — % abundance, standard-error of the mean (SEM), average abundance ratios and the number of positive spectral matches (PSMs) for proteins associating with CCR and CCR3A resin are listed. (DOCX) [file pgen.1008477.s009.docx]

**S7 Table**. Quantitative MS to compare the binding proteins of the wild-type Ulp2-CCR and the Ulp2-CCR^3A^ resins, using SF9-insect cell extracts expressing the yeast CMM complex. % abundance, standard-error of the mean (SEM), average abundance ratios and the number of positive spectral matches (PSMs) for proteins associating with CCR and CCR^3A^ resin are listed.

| Systematic  Name | % AbundanCe Ulp2-CCR | SEM of Ulp2-CCR % abundance | % Abundance Ulp2-CCR^3A^ | | SEM of Ulp2-CCR^3A^ % abundance | Ulp2-CCR/CCR^3A^ | | #  of PSMs | |
| --- | --- | --- | --- | --- | --- | --- | --- | --- | --- |
| YPR046W  (Mcm16) | 78.2% | 2.0% | 21.8% | | 2.0% | | 3.59 | | 16 |
| YLR381W  (Ctf3) | 75.4% | 2.0% | 24.6% | | 2.0% | | 3.07 | | 47 |
| YJR135C  (Mcm22) | 78.2% | 1.0% | 21.8% | | 1.0% | | 3.59 | | 56 |
| YIL031W  (Ulp2) | 37.7% | 0.0% | 62.3% | | 0.0% | | 0.61 | | 556 |
| S4WXF0 | 51.5% | 1.0% | 48.5% | | 1.0% | | 1.06 | | 13 |
| Q964Q9 | 50.6% | 1.0% | 49.4% | | 1.0% | | 1.02 | | 3 |
| Q963B7 | 51.5% | 1.0% | 48.5% | | 1.0% | | 1.06 | | 12 |
| Q962Y9 | 48.8% | 1.0% | 51.2% | | 1.0% | | 0.95 | | 21 |
| Q962Y8 | 49.7% | 1.0% | 50.3% | | 1.0% | | 0.99 | | 26 |
| Q962U2 | 49.7% | 1.0% | 50.3% | | 1.0% | | 0.99 | | 15 |
| Q962U1 | 50.6% | 1.0% | 49.4% | | 1.0% | | 1.02 | | 28 |
| Q962U0 | 51.5% | 0.0% | 48.5% | | 0.0% | | 1.06 | | 20 |
| Q962T8 | 51.5% | 1.0% | 48.5% | | 1.0% | | 1.06 | | 5 |
| Q962T6 | 52.4% | 1.0% | 47.6% | | 1.0% | | 1.10 | | 12 |
| Q962T5 | 52.4% | 2.0% | 47.6% | | 2.0% | | 1.10 | | 6 |
| Q962T3 | 48.8% | 2.0% | 51.2% | | 2.0% | | 0.95 | | 5 |
| Q962T2 | 50.6% | 1.0% | 49.4% | | 1.0% | | 1.02 | | 11 |
| Q962T1 | 47.8% | 1.0% | 52.2% | | 1.0% | | 0.92 | | 10 |
| Q962S9 | 49.7% | 1.0% | 50.3% | | 1.0% | | 0.99 | | 14 |
| Q962S8 | 50.6% | 1.0% | 49.4% | | 1.0% | | 1.02 | | 14 |
| Q962S7 | 50.6% | 1.0% | 49.4% | | 1.0% | | 1.02 | | 9 |
| Q962S6 | 52.4% | 1.0% | 47.6% | | 1.0% | | 1.10 | | 7 |
| Q962S3 | 37.7% | 0.0% | 62.3% | | 0.0% | | 0.61 | | 6 |
| Q962S0 | 49.7% | 1.0% | 50.3% | | 1.0% | | 0.99 | | 11 |
| Q962R9 | 55.2% | 0.0% | 44.8% | | 0.0% | | 1.23 | | 21 |
| Q962R7 | 53.4% | 2.0% | 46.6% | | 2.0% | | 1.14 | | 5 |
| Q962R6 | 53.4% | 0.0% | 46.6% | | 0.0% | | 1.14 | | 26 |
| Q962R5 | 48.8% | 0.0% | 51.2% | | 0.0% | | 0.95 | | 22 |
| Q962R4 | 53.4% | 1.0% | 46.6% | | 1.0% | | 1.14 | | 16 |
| Q962R2 | 52.4% | 0.0% | 47.6% | | 0.0% | | 1.10 | | 24 |
| Q962R1 | 55.2% | 1.0% | 44.8% | | 1.0% | | 1.23 | | 30 |
| Q962R0 | 57.0% | 0.0% | 43.0% | | 0.0% | | 1.33 | | 12 |
| Q962Q9 | 51.5% | 0.0% | 48.5% | | 0.0% | | 1.06 | | 13 |
| Q962Q8 | 54.3% | 1.0% | 45.7% | | 1.0% | | 1.19 | | 6 |
| Q962Q7 | 50.6% | 1.0% | 49.4% | | 1.0% | | 1.02 | | 23 |
| Q962Q6 | 54.3% | 0.0% | 45.7% | | 0.0% | | 1.19 | | 7 |
| Q962Q3 | 42.3% | 1.0% | 57.7% | | 1.0% | | 0.73 | | 10 |
| Q962Q2 | 55.2% | 1.0% | 44.8% | | 1.0% | | 1.23 | | 10 |
| Q962Q1 | 53.4% | 1.0% | 46.6% | | 1.0% | | 1.14 | | 7 |
| Q95V39 | 49.7% | 0.0% | 50.3% | | 0.0% | | 0.99 | | 40 |
| Q95V38 | 52.4% | 1.0% | 47.6% | | 1.0% | | 1.10 | | 14 |
| Q95V35 | 47.8% | 0.0% | 52.2% | | 0.0% | | 0.92 | | 49 |
| Q95V34 | 53.4% | 1.0% | 46.6% | | 1.0% | | 1.14 | | 26 |
| Q95V32 | 52.4% | 1.0% | 47.6% | | 1.0% | | 1.10 | | 30 |
| Q95V31 | 52.4% | 1.0% | 47.6% | | 1.0% | | 1.10 | | 27 |
| Q8WQJ2 | 52.4% | 1.0% | 47.6% | | 1.0% | | 1.10 | | 24 |
| Q8WQJ1 | 48.8% | 1.0% | 51.2% | | 1.0% | | 0.95 | | 14 |
| Q8WQJ0 | 51.5% | 1.0% | 48.5% | | 1.0% | | 1.06 | | 7 |
| Q8WQI9 | 48.8% | 1.0% | 51.2% | | 1.0% | | 0.95 | | 29 |
| Q8WQI7 | 51.5% | 1.0% | 48.5% | | 1.0% | | 1.06 | | 18 |
| Q8WQI6 | 50.6% | 1.0% | 49.4% | | 1.0% | | 1.02 | | 9 |
| Q8WQI5 | 51.5% | 0.0% | 48.5% | | 0.0% | | 1.06 | | 30 |
| Q8WQI3 | 52.4% | 1.0% | 47.6% | | 1.0% | | 1.10 | | 3 |
| Q8I9V9 | 51.5% | 0.0% | 48.5% | | 0.0% | | 1.06 | | 10 |
| Q8I865 | 53.4% | 0.0% | 46.6% | | 0.0% | | 1.14 | | 30 |
| Q8I864 | 53.4% | 1.0% | 46.6% | | 1.0% | | 1.14 | | 15 |
| Q7KF90 | 51.5% | 1.0% | 48.5% | | 1.0% | | 1.06 | | 19 |
| Q7KF88 | 52.4% | 1.0% | 47.6% | | 1.0% | | 1.10 | | 11 |
| P68203 | 53.4% | 1.0% | 46.6% | | 1.0% | | 1.14 | | 10 |
| P58375 | 51.5% | 0.0% | 48.5% | | 0.0% | | 1.06 | | 10 |
| J9Z496 | 48.8% | 2.0% | 51.2% | | 2.0% | | 0.95 | | 4 |
| G3LZX7 | 47.8% | 1.0% | 52.2% | | 1.0% | | 0.92 | | 5 |
| A0A2H1X3S3 | 42.3% | 1.0% | 57.7% | | 1.0% | | 0.73 | | 14 |
| A0A2H1X3E5 | 49.7% | 2.0% | 50.3% | | 2.0% | | 0.99 | | 4 |
| A0A2H1X373 | 49.7% | 3.0% | 50.3% | | 3.0% | | 0.99 | | 6 |
| A0A2H1X361 | 46.0% | 1.0% | 54.0% | | 1.0% | | 0.85 | | 7 |
| A0A2H1X346 | 48.8% | 3.0% | 51.2% | | 3.0% | | 0.95 | | 6 |
| A0A2H1X344 | 51.5% | 1.0% | 48.5% | | 1.0% | | 1.06 | | 8 |
| A0A2H1X332 | 51.5% | 2.0% | 48.5% | | 2.0% | | 1.06 | | 4 |
| A0A2H1X328 | 49.7% | 1.0% | 50.3% | | 1.0% | | 0.99 | | 3 |
| A0A2H1X308 | 51.5% | 1.0% | 48.5% | | 1.0% | | 1.06 | | 5 |
| A0A2H1X2T3 | 54.3% | 1.0% | 45.7% | | 1.0% | | 1.19 | | 5 |
| A0A2H1X2R9 | 51.5% | 1.0% | 48.5% | | 1.0% | | 1.06 | | 6 |
| A0A2H1X298 | 48.8% | 1.0% | 51.2% | | 1.0% | | 0.95 | | 5 |
| A0A2H1X270 | 48.8% | 1.0% | 51.2% | | 1.0% | | 0.95 | | 13 |
| A0A2H1X1N6 | 52.4% | 2.0% | 47.6% | | 2.0% | | 1.10 | | 3 |
| A0A2H1X1I7 | 60.7% | 1.0% | 39.3% | | 1.0% | | 1.55 | | 8 |
| A0A2H1X176 | 49.7% | 0.0% | 50.3% | | 0.0% | | 0.99 | | 13 |
| A0A2H1X138 | 51.5% | 1.0% | 48.5% | | 1.0% | | 1.06 | | 6 |
| A0A2H1X100 | 59.8% | 1.0% | 40.2% | | 1.0% | | 1.49 | | 3 |
| A0A2H1X0Y7 | 56.1% | 1.0% | 43.9% | | 1.0% | | 1.28 | | 41 |
| A0A2H1X0T2 | 61.6% | 1.0% | 38.4% | | 1.0% | | 1.61 | | 9 |
| A0A2H1X0T0 | 46.9% | 2.0% | 53.1% | | 2.0% | | 0.88 | | 3 |
| A0A2H1X0F6 | 46.9% | 1.0% | 53.1% | | 1.0% | | 0.88 | | 6 |
| A0A2H1X0C1 | 56.1% | 1.0% | 43.9% | | 1.0% | | 1.28 | | 14 |
| A0A2H1X096 | 52.4% | 1.0% | 47.6% | | 1.0% | | 1.10 | | 4 |
| A0A2H1X088 | 50.6% | 1.0% | 49.4% | | 1.0% | | 1.02 | | 13 |
| A0A2H1X028 | 55.2% | 1.0% | 44.8% | | 1.0% | | 1.23 | | 7 |
| A0A2H1WZW5 | 52.4% | 1.0% | 47.6% | | 1.0% | | 1.10 | | 6 |
| A0A2H1WZT8 | 49.7% | 1.0% | 50.3% | | 1.0% | | 0.99 | | 16 |
| A0A2H1WZB6 | 46.0% | 1.0% | 54.0% | | 1.0% | | 0.85 | | 3 |
| A0A2H1WZ80 | 37.7% | 2.0% | 62.3% | | 2.0% | | 0.61 | | 3 |
| A0A2H1WYZ7 | 58.9% | 0.0% | 41.1% | | 0.0% | | 1.43 | | 28 |
| A0A2H1WYZ1 | 51.5% | 1.0% | 48.5% | | 1.0% | | 1.06 | | 3 |
| A0A2H1WYZ0 | 53.4% | 0.0% | 46.6% | | 0.0% | | 1.14 | | 106 |
| A0A2H1WYY2 | 48.8% | 1.0% | 51.2% | | 1.0% | | 0.95 | | 12 |
| A0A2H1WYX5 | 58.0% | 1.0% | 42.0% | | 1.0% | | 1.38 | | 10 |
| A0A2H1WYV6 | 48.8% | 0.0% | 51.2% | | 0.0% | | 0.95 | | 106 |
| A0A2H1WYV1 | 50.6% | 1.0% | 49.4% | | 1.0% | | 1.02 | | 9 |
| A0A2H1WYK3 | 46.9% | 1.0% | 53.1% | | 1.0% | | 0.88 | | 5 |
| A0A2H1WY01 | 46.9% | 3.0% | 53.1% | | 3.0% | | 0.88 | | 4 |
| A0A2H1WXD8 | 60.7% | 1.0% | 39.3% | | 1.0% | | 1.55 | | 7 |
| A0A2H1WX92 | 53.4% | 1.0% | 46.6% | | 1.0% | | 1.14 | | 4 |
| A0A2H1WX66 | 40.5% | 3.0% | 59.5% | | 3.0% | | 0.68 | | 4 |
| A0A2H1WX45 | 49.7% | 1.0% | 50.3% | | 1.0% | | 0.99 | | 13 |
| A0A2H1WX17 | 43.2% | 1.0% | 56.8% | | 1.0% | | 0.76 | | 56 |
| A0A2H1WWX8 | 46.9% | 1.0% | 53.1% | | 1.0% | | 0.88 | | 5 |
| A0A2H1WWS3 | 50.6% | 1.0% | 49.4% | | 1.0% | | 1.02 | | 12 |
| A0A2H1WWK8 | 51.5% | 1.0% | 48.5% | | 1.0% | | 1.06 | | 11 |
| A0A2H1WWK0 | 47.8% | 1.0% | 52.2% | | 1.0% | | 0.92 | | 23 |
| A0A2H1WWH2 | 57.0% | 3.0% | 43.0% | | 3.0% | | 1.33 | | 3 |
| A0A2H1WW70 | 48.8% | 1.0% | 51.2% | | 1.0% | | 0.95 | | 11 |
| A0A2H1WVV7 | 57.0% | 2.0% | 43.0% | | 2.0% | | 1.33 | | 13 |
| A0A2H1WVF2 | 50.6% | 1.0% | 49.4% | | 1.0% | | 1.02 | | 15 |
| A0A2H1WV13 | 50.6% | 1.0% | 49.4% | | 1.0% | | 1.02 | | 9 |
| A0A2H1WUU5 | 51.5% | 1.0% | 48.5% | | 1.0% | | 1.06 | | 13 |
| A0A2H1WUS7 | 54.3% | 2.0% | 45.7% | | 2.0% | | 1.19 | | 7 |
| A0A2H1WUN4 | 52.4% | 2.0% | 47.6% | | 2.0% | | 1.10 | | 5 |
| A0A2H1WUI0 | 47.8% | 1.0% | 52.2% | | 1.0% | | 0.92 | | 7 |
| A0A2H1WUG0 | 50.6% | 2.0% | 49.4% | | 2.0% | | 1.02 | | 5 |
| A0A2H1WU00 | 48.8% | 3.0% | 51.2% | | 3.0% | | 0.95 | | 4 |
| A0A2H1WTV5 | 52.4% | 3.0% | 47.6% | | 3.0% | | 1.10 | | 3 |
| A0A2H1WTQ8 | 53.4% | 0.0% | 46.6% | | 0.0% | | 1.14 | | 48 |
| A0A2H1WTK7 | 50.6% | 1.0% | 49.4% | | 1.0% | | 1.02 | | 21 |
| A0A2H1WTA8 | 60.7% | 3.0% | 39.3% | | 3.0% | | 1.55 | | 3 |
| A0A2H1WT53 | 48.8% | 1.0% | 51.2% | | 1.0% | | 0.95 | | 4 |
| A0A2H1WSZ0 | 49.7% | 1.0% | 50.3% | | 1.0% | | 0.99 | | 6 |
| A0A2H1WSU8 | 47.8% | 0.0% | 52.2% | | 0.0% | | 0.92 | | 39 |
| A0A2H1WSU5 | 49.7% | 1.0% | 50.3% | | 1.0% | | 0.99 | | 10 |
| A0A2H1WSN6 | 59.8% | 2.0% | 40.2% | | 2.0% | | 1.49 | | 3 |
| A0A2H1WSN1 | 56.1% | 1.0% | 43.9% | | 1.0% | | 1.28 | | 13 |
| A0A2H1WSM8 | 58.9% | 2.0% | 41.1% | | 2.0% | | 1.43 | | 7 |
| A0A2H1WSH8 | 48.8% | 1.0% | 51.2% | | 1.0% | | 0.95 | | 6 |
| A0A2H1WSH2 | 56.1% | 1.0% | 43.9% | | 1.0% | | 1.28 | | 3 |
| A0A2H1WSE1 | 50.6% | 1.0% | 49.4% | | 1.0% | | 1.02 | | 47 |
| A0A2H1WRX5 | 50.6% | 0.0% | 49.4% | | 0.0% | | 1.02 | | 7 |
| A0A2H1WRS6 | 50.6% | 1.0% | 49.4% | | 1.0% | | 1.02 | | 16 |
| A0A2H1WRS1 | 57.0% | 1.0% | 43.0% | | 1.0% | | 1.33 | | 7 |
| A0A2H1WRK1 | 56.1% | 1.0% | 43.9% | | 1.0% | | 1.28 | | 13 |
| A0A2H1WRJ9 | 44.2% | 1.0% | 55.8% | | 1.0% | | 0.79 | | 24 |
| A0A2H1WRH9 | 50.6% | 3.0% | 49.4% | | 3.0% | | 1.02 | | 3 |
| A0A2H1WQZ8 | 52.4% | 1.0% | 47.6% | | 1.0% | | 1.10 | | 18 |
| A0A2H1WQU6 | 49.7% | 3.0% | 50.3% | | 3.0% | | 0.99 | | 3 |
| A0A2H1WQI2 | 50.6% | 1.0% | 49.4% | | 1.0% | | 1.02 | | 7 |
| A0A2H1WQ28 | 49.7% | 2.0% | 50.3% | | 2.0% | | 0.99 | | 3 |
| A0A2H1WPK2 | 50.6% | 1.0% | 49.4% | | 1.0% | | 1.02 | | 9 |
| A0A2H1WPC5 | 60.7% | 2.0% | 39.3% | | 2.0% | | 1.55 | | 6 |
| A0A2H1WP35 | 46.9% | 2.0% | 53.1% | | 2.0% | | 0.88 | | 5 |
| A0A2H1WNN8 | 52.4% | 1.0% | 47.6% | | 1.0% | | 1.10 | | 11 |
| A0A2H1WNI4 | 38.6% | 2.0% | 61.4% | | 2.0% | | 0.63 | | 8 |
| A0A2H1WNG5 | 50.6% | 2.0% | 49.4% | | 2.0% | | 1.02 | | 7 |
| A0A2H1WNA3 | 49.7% | 1.0% | 50.3% | | 1.0% | | 0.99 | | 3 |
| A0A2H1WN88 | 53.4% | 1.0% | 46.6% | | 1.0% | | 1.14 | | 27 |
| A0A2H1WN59 | 52.4% | 1.0% | 47.6% | | 1.0% | | 1.10 | | 25 |
| A0A2H1WN37 | 66.2% | 2.0% | 33.8% | | 2.0% | | 1.96 | | 6 |
| A0A2H1WMY6 | 49.7% | 1.0% | 50.3% | | 1.0% | | 0.99 | | 6 |
| A0A2H1WMW3 | 50.6% | 2.0% | 49.4% | | 2.0% | | 1.02 | | 4 |
| A0A2H1WMG1 | 51.5% | 2.0% | 48.5% | | 2.0% | | 1.06 | | 7 |
| A0A2H1WME0 | 43.2% | 3.0% | 56.8% | | 3.0% | | 0.76 | | 3 |
| A0A2H1WMC8 | 50.6% | 1.0% | 49.4% | | 1.0% | | 1.02 | | 3 |
| A0A2H1WMB6 | 52.4% | 1.0% | 47.6% | | 1.0% | | 1.10 | | 4 |
| A0A2H1WM16 | 57.0% | 3.0% | 43.0% | | 3.0% | | 1.33 | | 9 |
| A0A2H1WLW7 | 48.8% | 1.0% | 51.2% | | 1.0% | | 0.95 | | 4 |
| A0A2H1WLU6 | 46.0% | 8.0% | 54.0% | | 8.0% | | 0.85 | | 5 |
| A0A2H1WLN3 | 57.0% | 3.0% | 43.0% | | 3.0% | | 1.33 | | 5 |
| A0A2H1WLL1 | 54.3% | 1.0% | 45.7% | | 1.0% | | 1.19 | | 9 |
| A0A2H1WLF0 | 50.6% | 3.0% | 49.4% | | 3.0% | | 1.02 | | 3 |
| A0A2H1WL85 | 54.3% | 1.0% | 45.7% | | 1.0% | | 1.19 | | 17 |
| A0A2H1WL04 | 51.5% | 1.0% | 48.5% | | 1.0% | | 1.06 | | 12 |
| A0A2H1WL01 | 49.7% | 1.0% | 50.3% | | 1.0% | | 0.99 | | 8 |
| A0A2H1WKY2 | 51.5% | 1.0% | 48.5% | | 1.0% | | 1.06 | | 36 |
| A0A2H1WKT6 | 50.6% | 0.0% | 49.4% | | 0.0% | | 1.02 | | 7 |
| A0A2H1WKE8 | 48.8% | 1.0% | 51.2% | | 1.0% | | 0.95 | | 5 |
| A0A2H1WKD0 | 47.8% | 1.0% | 52.2% | | 1.0% | | 0.92 | | 14 |
| A0A2H1WKA2 | 52.4% | 1.0% | 47.6% | | 1.0% | | 1.10 | | 8 |
| A0A2H1WK37 | 49.7% | 1.0% | 50.3% | | 1.0% | | 0.99 | | 11 |
| A0A2H1WK25 | 55.2% | 0.0% | 44.8% | | 0.0% | | 1.23 | | 3 |
| A0A2H1WJY7 | 47.8% | 1.0% | 52.2% | | 1.0% | | 0.92 | | 20 |
| A0A2H1WJW3 | 50.6% | 2.0% | 49.4% | | 2.0% | | 1.02 | | 3 |
| A0A2H1WJV0 | 46.0% | 1.0% | 54.0% | | 1.0% | | 0.85 | | 5 |
| A0A2H1WJP6 | 60.7% | 1.0% | 39.3% | | 1.0% | | 1.55 | | 32 |
| A0A2H1WIY1 | 46.0% | 4.0% | 54.0% | | 4.0% | | 0.85 | | 4 |
| A0A2H1WIL8 | 45.1% | 0.0% | 54.9% | | 0.0% | | 0.82 | | 20 |
| A0A2H1WI71 | 51.5% | 1.0% | 48.5% | | 1.0% | | 1.06 | | 10 |
| A0A2H1WHL4 | 48.8% | 1.0% | 51.2% | | 1.0% | | 0.95 | | 6 |
| A0A2H1WHJ0 | 40.5% | 1.0% | 59.5% | | 1.0% | | 0.68 | | 11 |
| A0A2H1WHD1 | 56.1% | 2.0% | 43.9% | | 2.0% | | 1.28 | | 9 |
| A0A2H1WHC5 | 48.8% | 2.0% | 51.2% | | 2.0% | | 0.95 | | 5 |
| A0A2H1WH12 | 45.1% | 1.0% | 54.9% | | 1.0% | | 0.82 | | 3 |
| A0A2H1WGW9 | 61.6% | 0.0% | 38.4% | | 0.0% | | 1.61 | | 7 |
| A0A2H1WGV2 | 48.8% | 1.0% | 51.2% | | 1.0% | | 0.95 | | 7 |
| A0A2H1WGM5 | 51.5% | 3.0% | 48.5% | | 3.0% | | 1.06 | | 4 |
| A0A2H1WGF3 | 49.7% | 3.0% | 50.3% | | 3.0% | | 0.99 | | 3 |
| A0A2H1WG90 | 49.7% | 1.0% | 50.3% | | 1.0% | | 0.99 | | 7 |
| A0A2H1WG78 | 51.5% | 1.0% | 48.5% | | 1.0% | | 1.06 | | 21 |
| A0A2H1WG64 | 55.2% | 1.0% | 44.8% | | 1.0% | | 1.23 | | 3 |
| A0A2H1WFZ5 | 48.8% | 2.0% | 51.2% | | 2.0% | | 0.95 | | 5 |
| A0A2H1WFS5 | 58.0% | 0.0% | 42.0% | | 0.0% | | 1.38 | | 85 |
| A0A2H1WFE5 | 49.7% | 1.0% | 50.3% | | 1.0% | | 0.99 | | 27 |
| A0A2H1WFD3 | 55.2% | 1.0% | 44.8% | | 1.0% | | 1.23 | | 12 |
| A0A2H1WFA2 | 44.2% | 0.0% | 55.8% | | 0.0% | | 0.79 | | 220 |
| A0A2H1WES3 | 52.4% | 1.0% | 47.6% | | 1.0% | | 1.10 | | 27 |
| A0A2H1WEQ9 | 57.0% | 1.0% | 43.0% | | 1.0% | | 1.33 | | 6 |
| A0A2H1WEN9 | 51.5% | 1.0% | 48.5% | | 1.0% | | 1.06 | | 14 |
| A0A2H1WEJ6 | 54.3% | 2.0% | 45.7% | | 2.0% | | 1.19 | | 5 |
| A0A2H1WE58 | 61.6% | 1.0% | 38.4% | | 1.0% | | 1.61 | | 7 |
| A0A2H1WDX4 | 50.6% | 2.0% | 49.4% | | 2.0% | | 1.02 | | 4 |
| A0A2H1WDM1 | 48.8% | 3.0% | 51.2% | | 3.0% | | 0.95 | | 4 |
| A0A2H1WDF9 | 57.0% | 4.0% | 43.0% | | 4.0% | | 1.33 | | 4 |
| A0A2H1WD76 | 52.4% | 1.0% | 47.6% | | 1.0% | | 1.10 | | 4 |
| A0A2H1WD75 | 46.0% | 2.0% | 54.0% | | 2.0% | | 0.85 | | 3 |
| A0A2H1WD00 | 56.1% | 2.0% | 43.9% | | 2.0% | | 1.28 | | 8 |
| A0A2H1WCW8 | 52.4% | 1.0% | 47.6% | | 1.0% | | 1.10 | | 11 |
| A0A2H1WCK4 | 42.3% | 0.0% | 57.7% | | 0.0% | | 0.73 | | 3 |
| A0A2H1WC68 | 50.6% | 1.0% | 49.4% | | 1.0% | | 1.02 | | 6 |
| A0A2H1WC57 | 48.8% | 1.0% | 51.2% | | 1.0% | | 0.95 | | 3 |
| A0A2H1WC32 | 47.8% | 1.0% | 52.2% | | 1.0% | | 0.92 | | 9 |
| A0A2H1WBT4 | 51.5% | 2.0% | 48.5% | | 2.0% | | 1.06 | | 8 |
| A0A2H1WBL6 | 52.4% | 0.0% | 47.6% | | 0.0% | | 1.10 | | 8 |
| A0A2H1WBL5 | 47.8% | 1.0% | 52.2% | | 1.0% | | 0.92 | | 6 |
| A0A2H1WBG9 | 51.5% | 1.0% | 48.5% | | 1.0% | | 1.06 | | 8 |
| A0A2H1WBG0 | 53.4% | 0.0% | 46.6% | | 0.0% | | 1.14 | | 40 |
| A0A2H1WB18 | 48.8% | 1.0% | 51.2% | | 1.0% | | 0.95 | | 3 |
| A0A2H1WAU5 | 54.3% | 1.0% | 45.7% | | 1.0% | | 1.19 | | 46 |
| A0A2H1WAS4 | 53.4% | 1.0% | 46.6% | | 1.0% | | 1.14 | | 52 |
| A0A2H1WAM2 | 48.8% | 1.0% | 51.2% | | 1.0% | | 0.95 | | 8 |
| A0A2H1WAA4 | 50.6% | 0.0% | 49.4% | | 0.0% | | 1.02 | | 48 |
| A0A2H1WA27 | 49.7% | 2.0% | 50.3% | | 2.0% | | 0.99 | | 3 |
| A0A2H1W9Q5 | 57.0% | 4.0% | 43.0% | | 4.0% | | 1.33 | | 3 |
| A0A2H1W9K6 | 43.2% | 0.0% | 56.8% | | 0.0% | | 0.76 | | 4 |
| A0A2H1W935 | 52.4% | 2.0% | 47.6% | | 2.0% | | 1.10 | | 3 |
| A0A2H1W8W9 | 51.5% | 1.0% | 48.5% | | 1.0% | | 1.06 | | 5 |
| A0A2H1W8U6 | 46.9% | 0.0% | 53.1% | | 0.0% | | 0.88 | | 18 |
| A0A2H1W8L9 | 56.1% | 1.0% | 43.9% | | 1.0% | | 1.28 | | 6 |
| A0A2H1W8G0 | 42.3% | 2.0% | 57.7% | | 2.0% | | 0.73 | | 3 |
| A0A2H1W8D6 | 58.9% | 1.0% | 41.1% | | 1.0% | | 1.43 | | 26 |
| A0A2H1W891 | 53.4% | 3.0% | 46.6% | | 3.0% | | 1.14 | | 3 |
| A0A2H1W7W9 | 47.8% | 2.0% | 52.2% | | 2.0% | | 0.92 | | 3 |
| A0A2H1W7T2 | 49.7% | 1.0% | 50.3% | | 1.0% | | 0.99 | | 4 |
| A0A2H1W7R8 | 45.1% | 0.0% | 54.9% | | 0.0% | | 0.82 | | 58 |
| A0A2H1W7H4 | 52.4% | 1.0% | 47.6% | | 1.0% | | 1.10 | | 7 |
| A0A2H1W7E3 | 54.3% | 0.0% | 45.7% | | 0.0% | | 1.19 | | 42 |
| A0A2H1W7D1 | 53.4% | 0.0% | 46.6% | | 0.0% | | 1.14 | | 16 |
| A0A2H1W6T4 | 64.4% | 0.0% | 35.6% | | 0.0% | | 1.81 | | 209 |
| A0A2H1W6Q2 | 49.7% | 1.0% | 50.3% | | 1.0% | | 0.99 | | 9 |
| A0A2H1W6H0 | 48.8% | 1.0% | 51.2% | | 1.0% | | 0.95 | | 4 |
| A0A2H1W6B2 | 55.2% | 6.0% | 44.8% | | 6.0% | | 1.23 | | 3 |
| A0A2H1W6A4 | 49.7% | 1.0% | 50.3% | | 1.0% | | 0.99 | | 3 |
| A0A2H1W5S1 | 51.5% | 1.0% | 48.5% | | 1.0% | | 1.06 | | 4 |
| A0A2H1W5M2 | 61.6% | 11.0% | 38.4% | | 11.0% | | 1.61 | | 3 |
| A0A2H1W5L4 | 53.4% | 1.0% | 46.6% | | 1.0% | | 1.14 | | 16 |
| A0A2H1W5F8 | 60.7% | 1.0% | 39.3% | | 1.0% | | 1.55 | | 3 |
| A0A2H1W5F4 | 49.7% | 2.0% | 50.3% | | 2.0% | | 0.99 | | 5 |
| A0A2H1W5E0 | 54.3% | 3.0% | 45.7% | | 3.0% | | 1.19 | | 5 |
| A0A2H1W564 | 47.8% | 1.0% | 52.2% | | 1.0% | | 0.92 | | 3 |
| A0A2H1W556 | 51.5% | 1.0% | 48.5% | | 1.0% | | 1.06 | | 3 |
| A0A2H1W527 | 50.6% | 2.0% | 49.4% | | 2.0% | | 1.02 | | 5 |
| A0A2H1W4V5 | 53.4% | 2.0% | 46.6% | | 2.0% | | 1.14 | | 6 |
| A0A2H1W4E2 | 54.3% | 2.0% | 45.7% | | 2.0% | | 1.19 | | 11 |
| A0A2H1W488 | 49.7% | 1.0% | 50.3% | | 1.0% | | 0.99 | | 10 |
| A0A2H1W3Z1 | 54.3% | 1.0% | 45.7% | | 1.0% | | 1.19 | | 20 |
| A0A2H1W3Q8 | 46.0% | 1.0% | 54.0% | | 1.0% | | 0.85 | | 3 |
| A0A2H1W3L5 | 44.2% | 1.0% | 55.8% | | 1.0% | | 0.79 | | 4 |
| A0A2H1W3H7 | 46.0% | 2.0% | 54.0% | | 2.0% | | 0.85 | | 4 |
| A0A2H1W3G1 | 53.4% | 3.0% | 46.6% | | 3.0% | | 1.14 | | 3 |
| A0A2H1W3F8 | 46.9% | 0.0% | 53.1% | | 0.0% | | 0.88 | | 80 |
| A0A2H1W3F7 | 60.7% | 1.0% | 39.3% | | 1.0% | | 1.55 | | 14 |
| A0A2H1W380 | 51.5% | 1.0% | 48.5% | | 1.0% | | 1.06 | | 13 |
| A0A2H1W2T3 | 52.4% | 0.0% | 47.6% | | 0.0% | | 1.10 | | 13 |
| A0A2H1W2N3 | 50.6% | 1.0% | 49.4% | | 1.0% | | 1.02 | | 15 |
| A0A2H1W2F5 | 54.3% | 3.0% | 45.7% | | 3.0% | | 1.19 | | 3 |
| A0A2H1W267 | 58.0% | 2.0% | 42.0% | | 2.0% | | 1.38 | | 13 |
| A0A2H1W248 | 49.7% | 1.0% | 50.3% | | 1.0% | | 0.99 | | 3 |
| A0A2H1W238 | 46.9% | 1.0% | 53.1% | | 1.0% | | 0.88 | | 5 |
| A0A2H1W232 | 50.6% | 0.0% | 49.4% | | 0.0% | | 1.02 | | 10 |
| A0A2H1W1Y1 | 57.0% | 1.0% | 43.0% | | 1.0% | | 1.33 | | 12 |
| A0A2H1W1X7 | 48.8% | 0.0% | 51.2% | | 0.0% | | 0.95 | | 27 |
| A0A2H1W1T9 | 51.5% | 1.0% | 48.5% | | 1.0% | | 1.06 | | 7 |
| A0A2H1W1I1 | 53.4% | 1.0% | 46.6% | | 1.0% | | 1.14 | | 8 |
| A0A2H1W166 | 47.8% | 1.0% | 52.2% | | 1.0% | | 0.92 | | 3 |
| A0A2H1W0W7 | 49.7% | 2.0% | 50.3% | | 2.0% | | 0.99 | | 5 |
| A0A2H1W0W3 | 55.2% | 1.0% | 44.8% | | 1.0% | | 1.23 | | 9 |
| A0A2H1W0W2 | 48.8% | 1.0% | 51.2% | | 1.0% | | 0.95 | | 16 |
| A0A2H1W0V4 | 59.8% | 2.0% | 40.2% | | 2.0% | | 1.49 | | 7 |
| A0A2H1W0Q6 | 50.6% | 1.0% | 49.4% | | 1.0% | | 1.02 | | 9 |
| A0A2H1W0M1 | 53.4% | 2.0% | 46.6% | | 2.0% | | 1.14 | | 8 |
| A0A2H1W0I7 | 52.4% | 2.0% | 47.6% | | 2.0% | | 1.10 | | 4 |
| A0A2H1W0H9 | 50.6% | 2.0% | 49.4% | | 2.0% | | 1.02 | | 4 |
| A0A2H1W0H4 | 50.6% | 0.0% | 49.4% | | 0.0% | | 1.02 | | 29 |
| A0A2H1W0E4 | 55.2% | 2.0% | 44.8% | | 2.0% | | 1.23 | | 8 |
| A0A2H1W0C1 | 51.5% | 3.0% | 48.5% | | 3.0% | | 1.06 | | 5 |
| A0A2H1W083 | 50.6% | 0.0% | 49.4% | | 0.0% | | 1.02 | | 3 |
| A0A2H1W034 | 52.4% | 2.0% | 47.6% | | 2.0% | | 1.10 | | 5 |
| A0A2H1W011 | 57.0% | 2.0% | 43.0% | | 2.0% | | 1.33 | | 5 |
| A0A2H1W000 | 58.0% | 1.0% | 42.0% | | 1.0% | | 1.38 | | 8 |
| A0A2H1VZS1 | 53.4% | 2.0% | 46.6% | | 2.0% | | 1.14 | | 6 |
| A0A2H1VZP7 | 52.4% | 0.0% | 47.6% | | 0.0% | | 1.10 | | 5 |
| A0A2H1VZL4 | 52.4% | 0.0% | 47.6% | | 0.0% | | 1.10 | | 4 |
| A0A2H1VZK5 | 52.4% | 3.0% | 47.6% | | 3.0% | | 1.10 | | 3 |
| A0A2H1VZI7 | 49.7% | 0.0% | 50.3% | | 0.0% | | 0.99 | | 5 |
| A0A2H1VZH2 | 48.8% | 1.0% | 51.2% | | 1.0% | | 0.95 | | 6 |
| A0A2H1VZ97 | 46.0% | 2.0% | 54.0% | | 2.0% | | 0.85 | | 10 |
| A0A2H1VZ80 | 43.2% | 1.0% | 56.8% | | 1.0% | | 0.76 | | 5 |
| A0A2H1VZ57 | 46.0% | 1.0% | 54.0% | | 1.0% | | 0.85 | | 4 |
| A0A2H1VZ37 | 50.6% | 1.0% | 49.4% | | 1.0% | | 1.02 | | 17 |
| A0A2H1VZ07 | 52.4% | 1.0% | 47.6% | | 1.0% | | 1.10 | | 20 |
| A0A2H1VYD9 | 47.8% | 1.0% | 52.2% | | 1.0% | | 0.92 | | 4 |
| A0A2H1VY86 | 50.6% | 2.0% | 49.4% | | 2.0% | | 1.02 | | 10 |
| A0A2H1VY06 | 50.6% | 1.0% | 49.4% | | 1.0% | | 1.02 | | 17 |
| A0A2H1VXQ4 | 53.4% | 1.0% | 46.6% | | 1.0% | | 1.14 | | 15 |
| A0A2H1VXJ0 | 53.4% | 1.0% | 46.6% | | 1.0% | | 1.14 | | 3 |
| A0A2H1VXE3 | 49.7% | 1.0% | 50.3% | | 1.0% | | 0.99 | | 13 |
| A0A2H1VX99 | 54.3% | 1.0% | 45.7% | | 1.0% | | 1.19 | | 12 |
| A0A2H1VX72 | 49.7% | 3.0% | 50.3% | | 3.0% | | 0.99 | | 3 |
| A0A2H1VWN8 | 53.4% | 1.0% | 46.6% | | 1.0% | | 1.14 | | 4 |
| A0A2H1VWK1 | 62.6% | 1.0% | 37.4% | | 1.0% | | 1.67 | | 9 |
| A0A2H1VWE7 | 47.8% | 1.0% | 52.2% | | 1.0% | | 0.92 | | 7 |
| A0A2H1VWC5 | 46.0% | 2.0% | 54.0% | | 2.0% | | 0.85 | | 4 |
| A0A2H1VW71 | 44.2% | 1.0% | 55.8% | | 1.0% | | 0.79 | | 8 |
| A0A2H1VW38 | 57.0% | 1.0% | 43.0% | | 1.0% | | 1.33 | | 16 |
| A0A2H1VW16 | 51.5% | 1.0% | 48.5% | | 1.0% | | 1.06 | | 5 |
| A0A2H1VVM9 | 45.1% | 1.0% | 54.9% | | 1.0% | | 0.82 | | 3 |
| A0A2H1VVK9 | 56.1% | 1.0% | 43.9% | | 1.0% | | 1.28 | | 4 |
| A0A2H1VVJ7 | 48.8% | 1.0% | 51.2% | | 1.0% | | 0.95 | | 14 |
| A0A2H1VV87 | 56.1% | 1.0% | 43.9% | | 1.0% | | 1.28 | | 12 |
| A0A2H1VV46 | 58.0% | 3.0% | 42.0% | | 3.0% | | 1.38 | | 7 |
| A0A2H1VUX6 | 47.8% | 2.0% | 52.2% | | 2.0% | | 0.92 | | 3 |
| A0A2H1VUL2 | 57.0% | 8.0% | 43.0% | | 8.0% | | 1.33 | | 3 |
| A0A2H1VUB9 | 46.0% | 1.0% | 54.0% | | 1.0% | | 0.85 | | 3 |
| A0A2H1VU96 | 55.2% | 1.0% | 44.8% | | 1.0% | | 1.23 | | 8 |
| A0A2H1VTV2 | 46.0% | 0.0% | 54.0% | | 0.0% | | 0.85 | | 216 |
| A0A2H1VTQ6 | 53.4% | 1.0% | 46.6% | | 1.0% | | 1.14 | | 15 |
| A0A2H1VT34 | 53.4% | 1.0% | 46.6% | | 1.0% | | 1.14 | | 5 |
| A0A2H1VSY8 | 51.5% | 1.0% | 48.5% | | 1.0% | | 1.06 | | 4 |
| A0A2H1VSN4 | 47.8% | 2.0% | 52.2% | | 2.0% | | 0.92 | | 4 |
| A0A2H1VSL0 | 57.0% | 2.0% | 43.0% | | 2.0% | | 1.33 | | 4 |
| A0A2H1VSK3 | 52.4% | 1.0% | 47.6% | | 1.0% | | 1.10 | | 5 |
| A0A2H1VS21 | 45.1% | 2.0% | 54.9% | | 2.0% | | 0.82 | | 4 |
| A0A2H1VRL9 | 43.2% | 0.0% | 56.8% | | 0.0% | | 0.76 | | 57 |
| A0A2H1VRL4 | 52.4% | 1.0% | 47.6% | | 1.0% | | 1.10 | | 5 |
| A0A2H1VRI6 | 59.8% | 3.0% | 40.2% | | 3.0% | | 1.49 | | 10 |
| A0A2H1VRG3 | 46.0% | 2.0% | 54.0% | | 2.0% | | 0.85 | | 3 |
| A0A2H1VRE1 | 50.6% | 0.0% | 49.4% | | 0.0% | | 1.02 | | 17 |
| A0A2H1VQC5 | 51.5% | 5.0% | 48.5% | | 5.0% | | 1.06 | | 3 |
| A0A2H1VQ91 | 60.7% | 1.0% | 39.3% | | 1.0% | | 1.55 | | 13 |
| A0A2H1VQ03 | 47.8% | 4.0% | 52.2% | | 4.0% | | 0.92 | | 3 |
| A0A2H1VPN0 | 51.5% | 1.0% | 48.5% | | 1.0% | | 1.06 | | 6 |
| A0A2H1VPE5 | 56.1% | 5.0% | 43.9% | | 6.0% | | 1.28 | | 4 |
| A0A2H1VNW0 | 43.2% | 1.0% | 56.8% | | 1.0% | | 0.76 | | 6 |
| A0A2H1VNU9 | 54.3% | 1.0% | 45.7% | | 1.0% | | 1.19 | | 6 |
| A0A2H1VNQ1 | 47.8% | 1.0% | 52.2% | | 1.0% | | 0.92 | | 6 |
| A0A2H1VN08 | 49.7% | 1.0% | 50.3% | | 1.0% | | 0.99 | | 6 |
| A0A2H1VMV9 | 49.7% | 1.0% | 50.3% | | 1.0% | | 0.99 | | 15 |
| A0A2H1VMT9 | 50.6% | 3.0% | 49.4% | | 3.0% | | 1.02 | | 5 |
| A0A2H1VMM4 | 46.0% | 1.0% | 54.0% | | 1.0% | | 0.85 | | 29 |
| A0A2H1VMH7 | 47.8% | 0.0% | 52.2% | | 0.0% | | 0.92 | | 3 |
| A0A2H1VM88 | 53.4% | 1.0% | 46.6% | | 1.0% | | 1.14 | | 3 |
| A0A2H1VM80 | 56.1% | 3.0% | 43.9% | | 3.0% | | 1.28 | | 7 |
| A0A2H1VLL7 | 59.8% | 2.0% | 40.2% | | 2.0% | | 1.49 | | 8 |
| A0A2H1VLE5 | 46.9% | 2.0% | 53.1% | | 2.0% | | 0.88 | | 4 |
| A0A2H1VLC4 | 54.3% | 1.0% | 45.7% | | 1.0% | | 1.19 | | 8 |
| A0A2H1VL54 | 51.5% | 1.0% | 48.5% | | 1.0% | | 1.06 | | 10 |
| A0A2H1VL06 | 55.2% | 1.0% | 44.8% | | 1.0% | | 1.23 | | 13 |
| A0A2H1VKY3 | 51.5% | 1.0% | 48.5% | | 1.0% | | 1.06 | | 3 |
| A0A2H1VKV2 | 50.6% | 1.0% | 49.4% | | 1.0% | | 1.02 | | 9 |
| A0A2H1VKN9 | 47.8% | 1.0% | 52.2% | | 1.0% | | 0.92 | | 4 |
| A0A2H1VKL2 | 49.7% | 1.0% | 50.3% | | 1.0% | | 0.99 | | 13 |
| A0A2H1VKI2 | 47.8% | 2.0% | 52.2% | | 2.0% | | 0.92 | | 3 |
| A0A2H1VKG6 | 48.8% | 3.0% | 51.2% | | 3.0% | | 0.95 | | 3 |
| A0A2H1VJZ5 | 54.3% | 2.0% | 45.7% | | 2.0% | | 1.19 | | 8 |
| A0A2H1VJB7 | 56.1% | 2.0% | 43.9% | | 2.0% | | 1.28 | | 10 |
| A0A2H1VJ91 | 62.6% | 1.0% | 37.4% | | 1.0% | | 1.67 | | 8 |
| A0A2H1VJ55 | 58.0% | 1.0% | 42.0% | | 1.0% | | 1.38 | | 16 |
| A0A2H1VJ16 | 57.0% | 2.0% | 43.0% | | 2.0% | | 1.33 | | 10 |
| A0A2H1VIU1 | 52.4% | 2.0% | 47.6% | | 2.0% | | 1.10 | | 3 |
| A0A2H1VIP1 | 48.8% | 1.0% | 51.2% | | 1.0% | | 0.95 | | 13 |
| A0A2H1VIK8 | 52.4% | 1.0% | 47.6% | | 1.0% | | 1.10 | | 6 |
| A0A2H1VIJ5 | 51.5% | 2.0% | 48.5% | | 2.0% | | 1.06 | | 4 |
| A0A2H1VII9 | 51.5% | 1.0% | 48.5% | | 1.0% | | 1.06 | | 10 |
| A0A2H1VIF9 | 50.6% | 3.0% | 49.4% | | 3.0% | | 1.02 | | 4 |
| A0A2H1VIA1 | 49.7% | 1.0% | 50.3% | | 1.0% | | 0.99 | | 9 |
| A0A2H1VHH7 | 51.5% | 0.0% | 48.5% | | 0.0% | | 1.06 | | 11 |
| A0A2H1VHH6 | 52.4% | 1.0% | 47.6% | | 1.0% | | 1.10 | | 17 |
| A0A2H1VH93 | 50.6% | 4.0% | 49.4% | | 4.0% | | 1.02 | | 4 |
| A0A2H1VH62 | 47.8% | 1.0% | 52.2% | | 1.0% | | 0.92 | | 7 |
| A0A2H1VH60 | 49.7% | 1.0% | 50.3% | | 1.0% | | 0.99 | | 6 |
| A0A2H1VH44 | 46.9% | 1.0% | 53.1% | | 1.0% | | 0.88 | | 6 |
| A0A2H1VH29 | 48.8% | 0.0% | 51.2% | | 0.0% | | 0.95 | | 6 |
| A0A2H1VH22 | 48.8% | 1.0% | 51.2% | | 1.0% | | 0.95 | | 6 |
| A0A2H1VGV3 | 56.1% | 4.0% | 43.9% | | 4.0% | | 1.28 | | 3 |
| A0A2H1VGS2 | 32.2% | 13.0% | 67.8% | | 13.0% | | 0.47 | | 3 |
| A0A2H1VGH8 | 61.6% | 1.0% | 38.4% | | 1.0% | | 1.61 | | 10 |
| A0A2H1VGH6 | 50.6% | 1.0% | 49.4% | | 1.0% | | 1.02 | | 5 |
| A0A2H1VG69 | 58.0% | 1.0% | 42.0% | | 1.0% | | 1.38 | | 4 |
| A0A2H1VG27 | 46.9% | 2.0% | 53.1% | | 2.0% | | 0.88 | | 5 |
| A0A2H1VFY5 | 51.5% | 1.0% | 48.5% | | 1.0% | | 1.06 | | 8 |
| A0A2H1VFH2 | 52.4% | 4.0% | 47.6% | | 4.0% | | 1.10 | | 7 |
| A0A2H1VFF3 | 53.4% | 2.0% | 46.6% | | 2.0% | | 1.14 | | 11 |
| A0A2H1VFC7 | 49.7% | 1.0% | 50.3% | | 1.0% | | 0.99 | | 8 |
| A0A2H1VF90 | 49.7% | 2.0% | 50.3% | | 2.0% | | 0.99 | | 6 |
| A0A2H1VF42 | 50.6% | 2.0% | 49.4% | | 2.0% | | 1.02 | | 4 |
| A0A2H1VF18 | 53.4% | 0.0% | 46.6% | | 0.0% | | 1.14 | | 95 |
| A0A2H1VF17 | 57.0% | 1.0% | 43.0% | | 1.0% | | 1.33 | | 5 |
| A0A2H1VEE6 | 54.3% | 1.0% | 45.7% | | 1.0% | | 1.19 | | 14 |
| A0A2H1VE87 | 51.5% | 1.0% | 48.5% | | 1.0% | | 1.06 | | 8 |
| A0A2H1VDY8 | 50.6% | 1.0% | 49.4% | | 1.0% | | 1.02 | | 7 |
| A0A2H1VDU5 | 44.2% | 1.0% | 55.8% | | 1.0% | | 0.79 | | 14 |
| A0A2H1VD13 | 48.8% | 0.0% | 51.2% | | 0.0% | | 0.95 | | 75 |
| A0A2H1VCV7 | 47.8% | 4.0% | 52.2% | | 4.0% | | 0.92 | | 3 |
| A0A2H1VCM4 | 55.2% | 1.0% | 44.8% | | 1.0% | | 1.23 | | 21 |
| A0A2H1VC75 | 53.4% | 1.0% | 46.6% | | 1.0% | | 1.14 | | 3 |
| A0A2H1VC46 | 50.6% | 2.0% | 49.4% | | 2.0% | | 1.02 | | 5 |
| A0A2H1VC02 | 41.4% | 4.0% | 58.6% | | 4.0% | | 0.71 | | 5 |
| A0A2H1VBJ3 | 51.5% | 1.0% | 48.5% | | 1.0% | | 1.06 | | 3 |
| A0A2H1VBA5 | 46.9% | 0.0% | 53.1% | | 0.0% | | 0.88 | | 314 |
| A0A2H1VB61 | 59.8% | 0.0% | 40.2% | | 0.0% | | 1.49 | | 4 |
| A0A2H1VB60 | 47.8% | 2.0% | 52.2% | | 2.0% | | 0.92 | | 5 |
| A0A2H1VAU8 | 49.7% | 0.0% | 50.3% | | 0.0% | | 0.99 | | 3 |
| A0A2H1VAT6 | 51.5% | 1.0% | 48.5% | | 1.0% | | 1.06 | | 17 |
| A0A2H1VAM0 | 47.8% | 1.0% | 52.2% | | 1.0% | | 0.92 | | 4 |
| A0A2H1VAK4 | 52.4% | 1.0% | 47.6% | | 1.0% | | 1.10 | | 10 |
| A0A2H1VAD9 | 48.8% | 1.0% | 51.2% | | 1.0% | | 0.95 | | 33 |
| A0A2H1VAC7 | 49.7% | 2.0% | 50.3% | | 2.0% | | 0.99 | | 3 |
| A0A2H1VA67 | 55.2% | 1.0% | 44.8% | | 1.0% | | 1.23 | | 9 |
| A0A2H1V9Y9 | 61.6% | 1.0% | 38.4% | | 1.0% | | 1.61 | | 7 |
| A0A2H1V9R0 | 50.6% | 1.0% | 49.4% | | 1.0% | | 1.02 | | 3 |
| A0A2H1V9D5 | 48.8% | 1.0% | 51.2% | | 1.0% | | 0.95 | | 4 |
| A0A2H1V9C0 | 52.4% | 2.0% | 47.6% | | 2.0% | | 1.10 | | 5 |
| A0A2H1V9B8 | 57.0% | 2.0% | 43.0% | | 2.0% | | 1.33 | | 8 |
| A0A2H1V974 | 51.5% | 3.0% | 48.5% | | 3.0% | | 1.06 | | 3 |
| A0A2H1V8Y5 | 51.5% | 2.0% | 48.5% | | 2.0% | | 1.06 | | 6 |
| A0A2H1V8T3 | 57.0% | 2.0% | 43.0% | | 2.0% | | 1.33 | | 3 |
| A0A2H1V8D7 | 50.6% | 4.0% | 49.4% | | 4.0% | | 1.02 | | 3 |
| A0A2H1V8A4 | 46.0% | 1.0% | 54.0% | | 1.0% | | 0.85 | | 6 |
| A0A2H1V889 | 51.5% | 1.0% | 48.5% | | 1.0% | | 1.06 | | 4 |
| A0A2H1V870 | 50.6% | 1.0% | 49.4% | | 1.0% | | 1.02 | | 3 |
| A0A2H1V844 | 54.3% | 4.0% | 45.7% | | 4.0% | | 1.19 | | 4 |
| A0A2H1V837 | 51.5% | 1.0% | 48.5% | | 1.0% | | 1.06 | | 7 |
| A0A2H1V7K3 | 60.7% | 3.0% | 39.3% | | 3.0% | | 1.55 | | 3 |
| A0A2H1V755 | 62.6% | 1.0% | 37.4% | | 1.0% | | 1.67 | | 5 |
| A0A2H1V6X2 | 53.4% | 1.0% | 46.6% | | 1.0% | | 1.14 | | 11 |
| A0A2H1V6T9 | 53.4% | 2.0% | 46.6% | | 2.0% | | 1.14 | | 4 |
| A0A2H1V6T3 | 47.8% | 1.0% | 52.2% | | 1.0% | | 0.92 | | 6 |
| A0A2H1V6P2 | 48.8% | 1.0% | 51.2% | | 1.0% | | 0.95 | | 4 |
| A0A2H1V6F8 | 54.3% | 1.0% | 45.7% | | 1.0% | | 1.19 | | 8 |
| A0A2H1V672 | 60.7% | 1.0% | 39.3% | | 1.0% | | 1.55 | | 10 |
| A0A2H1V648 | 57.0% | 1.0% | 43.0% | | 1.0% | | 1.33 | | 10 |
| A0A2H1V632 | 45.1% | 0.0% | 54.9% | | 0.0% | | 0.82 | | 78 |
| A0A2H1V5U3 | 51.5% | 1.0% | 48.5% | | 1.0% | | 1.06 | | 10 |
| A0A2H1V5J4 | 51.5% | 2.0% | 48.5% | | 2.0% | | 1.06 | | 8 |
| A0A2H1V5H5 | 48.8% | 1.0% | 51.2% | | 1.0% | | 0.95 | | 3 |
| A0A2H1V5C0 | 49.7% | 1.0% | 50.3% | | 1.0% | | 0.99 | | 10 |
| A0A2H1V5B6 | 51.5% | 1.0% | 48.5% | | 1.0% | | 1.06 | | 11 |
| A0A2H1V535 | 54.3% | 1.0% | 45.7% | | 1.0% | | 1.19 | | 8 |
| A0A2H1V4Z6 | 54.3% | 2.0% | 45.7% | | 2.0% | | 1.19 | | 5 |
| A0A2H1V4N6 | 57.0% | 1.0% | 43.0% | | 1.0% | | 1.33 | | 4 |
| A0A2H1V4N3 | 51.5% | 2.0% | 48.5% | | 2.0% | | 1.06 | | 3 |
| A0A2H1V485 | 60.7% | 1.0% | 39.3% | | 1.0% | | 1.55 | | 21 |
| A0A2H1V442 | 52.4% | 1.0% | 47.6% | | 1.0% | | 1.10 | | 3 |
| A0A2H1V407 | 48.8% | 2.0% | 51.2% | | 2.0% | | 0.95 | | 3 |
| A0A2H1V3Y5 | 51.5% | 1.0% | 48.5% | | 1.0% | | 1.06 | | 3 |
| A0A2H1V3M7 | 58.9% | 1.0% | 41.1% | | 1.0% | | 1.43 | | 8 |
| A0A2H1V3C6 | 51.5% | 2.0% | 48.5% | | 2.0% | | 1.06 | | 3 |
| A0A2H1V392 | 50.6% | 0.0% | 49.4% | | 0.0% | | 1.02 | | 25 |
| A0A2H1V364 | 52.4% | 2.0% | 47.6% | | 2.0% | | 1.10 | | 4 |
| A0A2H1V357 | 58.0% | 2.0% | 42.0% | | 2.0% | | 1.38 | | 19 |
| A0A2H1V339 | 46.0% | 2.0% | 54.0% | | 2.0% | | 0.85 | | 5 |
| A0A2H1V338 | 50.6% | 1.0% | 49.4% | | 1.0% | | 1.02 | | 9 |
| A0A2H1V328 | 49.7% | 1.0% | 50.3% | | 1.0% | | 0.99 | | 10 |
| A0A2H1V327 | 51.5% | 2.0% | 48.5% | | 2.0% | | 1.06 | | 9 |
| A0A2H1V319 | 62.6% | 3.0% | 37.4% | | 3.0% | | 1.67 | | 4 |
| A0A2H1V301 | 47.8% | 1.0% | 52.2% | | 1.0% | | 0.92 | | 6 |
| A0A2H1V2Z5 | 39.6% | 1.0% | 60.4% | | 1.0% | | 0.65 | | 25 |
| A0A2H1V2Z4 | 59.8% | 0.0% | 40.2% | | 0.0% | | 1.49 | | 3 |
| A0A2H1V2P2 | 53.4% | 1.0% | 46.6% | | 1.0% | | 1.14 | | 9 |
| A0A2H1V2N8 | 42.3% | 2.0% | 57.7% | | 2.0% | | 0.73 | | 5 |
| A0A2H1V2N3 | 57.0% | 1.0% | 43.0% | | 1.0% | | 1.33 | | 5 |
| A0A2H1V2L2 | 53.4% | 2.0% | 46.6% | | 2.0% | | 1.14 | | 9 |
| A0A2H1V2G5 | 54.3% | 1.0% | 45.7% | | 1.0% | | 1.19 | | 4 |
| A0A2H1V2G3 | 49.7% | 1.0% | 50.3% | | 1.0% | | 0.99 | | 21 |
| A0A2H1V2E8 | 50.6% | 0.0% | 49.4% | | 0.0% | | 1.02 | | 14 |
| A0A2H1V2E1 | 52.4% | 0.0% | 47.6% | | 0.0% | | 1.10 | | 3 |
| A0A2H1V2D8 | 59.8% | 1.0% | 40.2% | | 1.0% | | 1.49 | | 17 |
| A0A2H1V244 | 50.6% | 1.0% | 49.4% | | 1.0% | | 1.02 | | 7 |
| A0A2H1V1E5 | 56.1% | 1.0% | 43.9% | | 1.0% | | 1.28 | | 24 |
| A0A2H1V1A5 | 51.5% | 1.0% | 48.5% | | 1.0% | | 1.06 | | 3 |
| A0A2H1V154 | 48.8% | 0.0% | 51.2% | | 0.0% | | 0.95 | | 5 |
| A0A2H1V151 | 51.5% | 3.0% | 48.5% | | 3.0% | | 1.06 | | 3 |
| A0A2H1V148 | 47.8% | 1.0% | 52.2% | | 1.0% | | 0.92 | | 4 |
| A0A2H1V100 | 52.4% | 4.0% | 47.6% | | 4.0% | | 1.10 | | 3 |
| A0A2H1V0Y7 | 49.7% | 2.0% | 50.3% | | 2.0% | | 0.99 | | 3 |
| A0A2H1V0P5 | 54.3% | 1.0% | 45.7% | | 1.0% | | 1.19 | | 3 |
| A0A2H1V0N7 | 58.0% | 1.0% | 42.0% | | 1.0% | | 1.38 | | 23 |
| A0A2H1V0L1 | 52.4% | 2.0% | 47.6% | | 2.0% | | 1.10 | | 3 |
| A0A2H1V073 | 55.2% | 0.0% | 44.8% | | 0.0% | | 1.23 | | 7 |
| A0A2H1UZY3 | 49.7% | 1.0% | 50.3% | | 1.0% | | 0.99 | | 5 |
| A0A2H1UZX1 | 52.4% | 3.0% | 47.6% | | 3.0% | | 1.10 | | 3 |
| A0A2H1UZV5 | 66.2% | 1.0% | 33.8% | | 1.0% | | 1.96 | | 8 |
| A0A1W6S692 | 51.5% | 1.0% | 48.5% | | 1.0% | | 1.06 | | 6 |
| A0A1V0JHV2 | 57.0% | 2.0% | 43.0% | | 2.0% | | 1.33 | | 3 |
| A0A1V0JHU5 | 58.9% | 2.0% | 41.1% | | 2.0% | | 1.43 | | 4 |
| A0A1V0JHU4 | 48.8% | 1.0% | 51.2% | | 1.0% | | 0.95 | | 3 |
| A0A1S6Q5K6 | 47.8% | 2.0% | 52.2% | | 2.0% | | 0.92 | | 3 |
| A0A1P8W8N0 | 53.4% | 0.0% | 46.6% | | 0.0% | | 1.14 | | 44 |
| A0A1C7D1B9 | 52.4% | 0.0% | 47.6% | | 0.0% | | 1.10 | | 4 |
| A0A0R5RHL7 | 54.3% | 1.0% | 45.7% | | 1.0% | | 1.19 | | 9 |
| A0A0K2CU30 | 49.7% | 1.0% | 50.3% | | 1.0% | | 0.99 | | 5 |
| A0A0K2CTV2 | 51.5% | 1.0% | 48.5% | | 1.0% | | 1.06 | | 7 |
| A0A0K2CTV1 | 52.4% | 1.0% | 47.6% | | 1.0% | | 1.10 | | 22 |
| A0A0K2CTM7 | 45.1% | 1.0% | 54.9% | | 1.0% | | 0.82 | | 7 |
| A0A0K2CTM2 | 50.6% | 0.0% | 49.4% | | 0.0% | | 1.02 | | 28 |
| A0A0K0K804 | 52.4% | 0.0% | 47.6% | | 0.0% | | 1.10 | | 4 |
| A0A0K0K6W5 | 55.2% | 0.0% | 44.8% | | 0.0% | | 1.23 | | 86 |
| A0A0K0K6V6 | 57.0% | 1.0% | 43.0% | | 1.0% | | 1.33 | | 13 |
| A0A096UX67 | 52.4% | 2.0% | 47.6% | 2.0% | | 1.10 | | 6 | |
