# Supplementary material for: Recruitment of the Ulp2 protease to the inner kinetochore prevents its hyper-sumoylation to ensure accurate chromosome segregation
Source: PLoS Genet. 2019 Nov 20;15(11):e1008477. doi: 10.1371/journal.pgen.1008477 (PMC6892545; doi:10.1371/journal.pgen.1008477)
Supplement: S8 Table — Median chromosome III loss rates, the 95% confidence interval (CI) and the Fold-change relative to WT are shown. (DOCX) [file pgen.1008477.s010.docx]

**S8 Table**. Quantitative mating results for assaying the rate of chromosome III loss, which are used to generate Figures 5B, 6A and 7A. Median chromosome III loss rates, the 95% confidence interval (CI) and the Fold-change relative to WT are shown.

| Genotype | Median | 95% CI Upper Limit | 95% CI Lower Limit | Fold-change to WT |
| --- | --- | --- | --- | --- |
| WT | 4.82E-07 | 6.19E-07 | 3.90E-07 | 1 |
| *SIM^3A^* | 3.70E-06 | 4.92E-06 | 2.17E-06 | 8 |
| *CCR^3A^* | 2.10E-06 | 2.73E-06 | 1.33E-06 | 4 |
| *mcm16Δ* | 2.03E-06 | 4.40E-06 | 6.06E-07 | 4 |
| *SIM^3A^CCR^3A^* | 3.08E-05 | 3.31E-05 | 2.19E-05 | 64 |
| *SIM^3A^mcm16Δ* | 2.50E-06 | 3.79E-06 | 1.72E-06 | 5 |
| *CCR^3A^mcm16Δ* | 1.58E-06 | 2.07E-06 | 9.52E-07 | 3 |
| *SIM^3A^CCR^3A^mcm16Δ* | 3.03E-06 | 3.72E-06 | 1.06E-06 | 6 |
| *smt3allR* | 2.76E-05 | 3.59E-05 | 2.19E-05 | 57 |
| *smt3allR SIM^3A^CCR^3A^* | 2.88E-06 | 3.69E-06 | 1.58E-06 | 6 |
