# Supplementary material for: Recruitment of the Ulp2 protease to the inner kinetochore prevents its hyper-sumoylation to ensure accurate chromosome segregation
Source: PLoS Genet. 2019 Nov 20;15(11):e1008477. doi: 10.1371/journal.pgen.1008477 (PMC6892545; doi:10.1371/journal.pgen.1008477)
Supplement: S10 Table — (DOCX) [file pgen.1008477.s012.docx]

**S10 Table**. Quantitative MS to compare the % abundance of proteins expressed on each chromosome in wild-type and the *ulp2-SIM^3A^CCR^3A^* mutants.

| Chr # | % abundance *ulp2-SIM^3A^ CCR^3A^* #1 | % abundance WT #1 | % abundance WT #2 | % abundance *ulp2-SIM^3A^ CCR^3A^* #2 |
| --- | --- | --- | --- | --- |
| I | 24.4% | 24.0% | 25.7% | 26.1% |
| II | 25.0% | 23.4% | 25.5% | 25.9% |
| III | 25.0% | 23.8% | 25.3% | 25.8% |
| IV | 24.9% | 23.6% | 25.6% | 25.9% |
| V | 24.9% | 23.9% | 25.5% | 25.8% |
| VI | 24.9% | 23.4% | 25.9% | 25.6% |
| VII | 24.7% | 24.0% | 25.2% | 26.0% |
| VIII | 24.7% | 24.1% | 25.0% | 26.1% |
| IX | 24.8% | 23.7% | 25.8% | 25.8% |
| X | 24.7% | 24.1% | 25.1% | 26.0% |
| XI | 25.1% | 23.6% | 25.8% | 25.6% |
| XII | 24.7% | 23.9% | 25.4% | 26.0% |
| XIII | 25.1% | 23.7% | 25.5% | 25.6% |
| XIV | 24.9% | 23.8% | 25.5% | 25.7% |
| XV | 24.9% | 23.6% | 25.6% | 25.8% |
| XVI | 24.9% | 23.8% | 25.1% | 26.2% |
