# Supplementary material for: Recruitment of the Ulp2 protease to the inner kinetochore prevents its hyper-sumoylation to ensure accurate chromosome segregation
Source: PLoS Genet. 2019 Nov 20;15(11):e1008477. doi: 10.1371/journal.pgen.1008477 (PMC6892545; doi:10.1371/journal.pgen.1008477)
Supplement: S11 Table — (DOCX) [file pgen.1008477.s013.docx]

**S11 Table**. Quantitative MS to compare the % abundance of proteins expressed on each chromosome in wild-type and several independently prepared *ulp2Δ* mutants.

| Chr  # | % WT | % *ulp2∆* #1 | % *ulp2∆* #2 | % *ulp2∆* #3 | % *ulp2∆* #4 |
| --- | --- | --- | --- | --- | --- |
| I | 15.6% | 21.3% | 21.2% | 23.9% | 23.1% |
| II | 21.8% | 18.3% | 17.8% | 19.9% | 19.4% |
| III | 16.0% | 21.6% | 22.0% | 22.4% | 22.8% |
| IV | 21.4% | 19.3% | 18.3% | 20.1% | 20.0% |
| V | 19.2% | 19.9% | 19.4% | 21.3% | 21.1% |
| VI | 22.0% | 18.2% | 17.8% | 20.2% | 19.2% |
| VII | 21.1% | 19.2% | 18.8% | 20.8% | 20.2% |
| VIII | 22.3% | 19.0% | 18.2% | 19.8% | 18.7% |
| IX | 18.2% | 21.0% | 20.2% | 21.9% | 21.8% |
| X | 18.9% | 20.2% | 19.3% | 22.2% | 21.2% |
| XI | 17.3% | 20.9% | 20.1% | 22.5% | 21.5% |
| XII | 22.8% | 18.2% | 17.7% | 19.5% | 19.0% |
| XIII | 22.3% | 19.3% | 17.8% | 19.3% | 19.3% |
| XIV | 19.2% | 19.9% | 19.8% | 21.8% | 20.9% |
| XV | 19.8% | 19.7% | 18.2% | 20.7% | 20.2% |
| XVI | 19.5% | 20.0% | 19.4% | 21.3% | 20.6% |
