# Supplementary material for: Recruitment of the Ulp2 protease to the inner kinetochore prevents its hyper-sumoylation to ensure accurate chromosome segregation
Source: PLoS Genet. 2019 Nov 20;15(11):e1008477. doi: 10.1371/journal.pgen.1008477 (PMC6892545; doi:10.1371/journal.pgen.1008477)
Supplement: S12 Table — (DOCX) [file pgen.1008477.s014.docx]

**S12 Table.** Yeast strains and plasmids used in this study

| Strain Name | Genotype | Source |
| --- | --- | --- |
| HZY2101 | MATa, *HF-SMT3* *sml1∆::TRP1 arg4∆ ura3-52 leu2∆1 trp1∆63 his3∆200 lys2∆Bgl hom3-10 ade2∆ ade8*. | Albuquerque, et al., PLoS genetics 2013 |
| HZY3963 | MATa, *ulp2-SIM^3A^-TAF:: kanMX6*, derived from HZY2101 | Albuquerque, et al., J Biol Chem 2018 |
| HZY3974 | MATa, *ulp2-CCR^3A^-TAF:: kanMX6*, derived from HZY2101 | This study |
| HZY3984 | MATa, *ulp2-SIM^3A^CCR^3A^-TAF:: kanMX6*, derived from HZY2101 | This study |
| HZY3752 | MATa, *ulp2Δ::natMX4*, isogenic to HZY2101 | This study |
| HZY1927 | MATα, *mcm16Δ::His3MX6 ulp2Δ::natMX4*, isogenic to HZY2101 | This study |
| W303-1a | MATa, *ade2-1 can1-100 his3-11, 15 leu2-3, 112 trp1-1 ura3-1 RAD5+* | This study |
| W303-1b | MATα, *ade2-1 can1-100 his3-11, 15 leu2-3, 112 trp1-1 ura3-1 RAD5+* | This study |
| HZY276 | W303, MATα, *Mcm21-3×FLAG-ProtA::His3MX6* | This study |
| HZY287 | W303, MATa, *Mcm21-3×FLAG-ProtA::His3MX6 ulp2-SIM^3A^::kanMX6* | This study |
| HZY289 | W303, MATa, *Mcm21-3×FLAG-ProtA::His3MX6 ulp2-CCR^3A^::kanMX6* | This study |
| HZY294 | W303, MATa, *Mcm21-3×FLAG-ProtA::His3MX6 ulp2-SIM^3A^CCR^3A^::kanMX6* | This study |
| HZY036 | W303, MATa, *Mcm16-TAF::kanMX6 ulp2Δ::natMX4* pRS316-*ULP2* | This study |
| HZY040 | W303, MATa, *Okp1-TAF::kanMX6 ulp2Δ::natMX4* pRS316-*ULP2* | This study |
| HZY027 | W303, MATa, *Ame1-TAF::kanMX6 ulp2Δ::natMX4* pRS316-*ULP2* | This study |
| HZY038 | W303, MATa, *Mcm22-TAF::kanMX6 ulp2Δ::natMX4* pRS316-*ULP2* | This study |
| HZY940 | W303, MATa, *Mcm21-TEV-ProtA::His3MX6* | This study |
| HZY944 | W303, MATa, *Mcm21-TEV-ProtA::His3MX6 mcm16Δ::kanMX6* | This study |
| HZY958 | W303, MATa, *Mcm21-TEV-ProtA::His3MX6 ctf19Δ::kanMX6* | This study |
| HZY1362 | W303, MATa, *Mcm21-TEV-ProtA::His3MX6 chl4Δ::hygro* | This study |
| HZY1363 | W303, MATa, *Mcm21-TEV-ProtA::His3MX6 cnn1Δ::hygro* | This study |
| HZY1360 | W303, MATa, *Mcm16-TAF::kanMX6 chl4Δ::hygro* | This study |
| HZY1361 | W303, MATa, *Mcm16-TAF::kanMX6 cnn1Δ::hygro* | This study |
| HZY1300 | W303, MATa, *CTF3-TAF::kanMX6* | This study |
| HZY1302 | W303, MATa, *Ctf3-TAF::kanMX6 mcm16Δ::hygro* | This study |
| HZY1305 | W303, MATa, *Ctf3-TAF::kanMX6 mcm21Δ::hygro* | This study |
| HZY1323 | W303, MATa, *Ctf3-TAF::kanMX6 mcm22Δ::hygro* | This study |
| HZY1312 | W303, MATα, *Mcm22-TAF::kanMX6* | This study |
| HZY1316 | W303, MATα, *Mcm22-TAF::kanMX6 mcm16Δ::hygro* | This study |
| HZY1314 | W303, MATα, *Mcm22-TAF::kanMX6 ctf3Δ::hygro* | This study |
| HZY1307 | W303, MATa, *Mcm16-TAF::kanMX6* | This study |
| HZY1310 | W303, MATa, *Mcm16-TAF::kanMX6 mcm22Δ::hygro* | This study |
| HZY1308 | W303, MATa, *Mcm16-TAF::kanMX6 ctf3Δ::hygro* | This study |
| HZY3545 | W303, MATa/MATα, *ULP2/ulp2-707Δ::hygro* | This study |
| HZY3547 | W303, MATa/MATα, *ULP2/ulp2-SIM^3A^CCR^3A^::kanMX6* | This study |
| HZY600 | MATα, *LEU2 ura3-52 leu2∆1 trp1∆63 his3∆200 lys2∆Bgl hom3-10 ade2∆1 ade8* | This study |
| HZY602 | MATα, *mad1∆:: His3MX6*, derived from HZY600 | This study |
| HZY632 | MATα, *ulp2-SIM^3A^:: kanMX6*, derived from HZY600 | This study |
| HZY633 | MATα, *ulp2-CCR^3A^:: kanMX6*, derived from HZY600 | This study |
| HZY607 | MATα, *ulp2-SIM^3A^CCR^3A^::kanMX6*, derived from HZY600 | This study |
| HZY848 | MATα, *mcm16Δ::kanMX6*, derived from HZY600 | This study |
| HZY652 | MATα, *mcm16Δ::natMX4* *ulp2-CCR^3A^:: kanMX6*, derived from HZY632 | This study |
| HZY872 | MATα, *mcm16Δ::natMX4* *ulp2-SIM^3A^:: kanMX6*, derived from HZY633 | This study |
| HZY866 | MATα, *mcm16Δ::natMX4* *ulp2-SIM^3A^CCR^3A^::kanMX6*, derived from HZY607 | This study |
| hzy611 | MATα, *smt3-allR::kanMX6*, derived from HZY600 | This study |
| HZY392 | MATα, *smt3-allR::kanMX6 ulp2-SIM^3A^CCR^3A^::NatMX4*, derived from HZY600 | This study |
| HZY393 | MATα, *smt3-allR::kanMX6 ulp2-SIM^3A^CCR^3A^::NatMX4*, derived from HZY600 | This study |
| HZY3658 | W303, MATa, *ulp2Δ::natMX4* pRS316-*ULP2* | This study |
| HZY846 | W303, MATa, *ulp2Δ::natMX4* *mcm16Δ::kanMX6* pRS316-*ULP2* | This study |
| HZY119 | W303, MATa, *Mcm21-TEV-ProtA::His3MX6 ulp2Δ::natMX4* | This study |
| HZY942 | W303, MATa, *Mcm21-TEV-ProtA::His3MX6 ulp2Δ::natMX4* *mcm16Δ::kanMX6* | This study |
| HZY1318 | W303, MATa, *Ame1-TAF::kanMX6* | This study |
| HZY1320 | W303, MATa, *Ame1-TAF::kanMX6 mcm16Δ::hygro* | This study |
| HZY028 | W303, MATa, *Ame1-TAF::kanMX6 ulp2Δ::natMX4* | This study |
| HZY1330 | W303, MATa, *Ame1-TAF::kanMX6 mcm16Δ::hygro ulp2Δ::natMX4* | This study |
| HZY318 | W303, MATα, *Mcm21-3×FLAG-ProtA::His3MX6 mcm16Δ::kanMX6*, derived from HZY276 | This study |
| HZY334 | MATα, *Mcm21-3×FLAG-ProtA::His3MX6 ulp2-SIM^3A^::kanMX6 mcm16Δ::natMX4*, derived from HZY287 | This study |
| HZY335 | MATα, *Mcm21-3×FLAG-ProtA::His3MX6 ulp2-CCR^3A^::kanMX6 mcm16Δ::natMX4*, derived from HZY289 | This study |
| HZY337 | MATa, *Mcm21-3×FLAG-ProtA::His3MX6 ulp2-SIM^3A^CCR^3A^::kanMX6 mcm16Δ::natMX4*, derived from HZY294 | This study |
| HZY4371 | W303, MATa, *Ame1-TAF::His3MX6* *ulp2Δ::natMX4* pRS316-*ULP2* | This study |
| HZY4372 | W303, MATa, *OKP1-TAF::His3MX6* *ulp2Δ::natMX4* pRS316-*ULP2* | This study |
| HZY4373 | W303, MATa, *MCM16-TAF::His3MX6* *ulp2Δ::natMX4* pRS316-*ULP2* | This study |
| HZY4379 | W303, MATa, *MCM22-TAF::His3MX6* *ulp2Δ::natMX4* pRS316-*ULP2* | This study |
| HZY118 | W303, MATa, *Mcm21-TEV-ProtA::His3MX6*, *ulp2Δ::natMX4* | This study |
| HZY4133 | W303, MATa, *smt3-allR::kanMX6 Ame1-TAF::His3MX6* *ulp2Δ::natMX4* | This study |
| HZY4134 | W303, MATa, *smt3-allR::kanMX6 OKP1-TAF::His3MX6* *ulp2Δ::natMX4* | This study |
| HZY4129 | W303, MATa, *smt3-allR::kanMX6 MCM16-TAF::His3MX6* *ulp2Δ::natMX4* | This study |
| HZY4131 | W303, MATa, *smt3-allR::kanMX6 MCM22-TAF::His3MX6* *ulp2Δ::natMX4* | This study |
| HZY4127 | W303, MATa, *smt3-allR::kanMX6 Mcm21-TEV-ProtA::His3MX6* *ulp2Δ::natMX4* | This study |
| SBY8361 | Dsn1-HIS-FLAG:URA3, *ndc10-1, bar1-1,* MAT a, W303 (*ura3-1, leu2-3,112, his3-11, trp1-1, can1-100, ade2-1*) | Lang J, et al. [eLife. 2018; 7: e37819.](https://www.ncbi.nlm.nih.gov/pmc/articles/PMC6097842/) |
| HZY299 | W303, MATα, *Mcm21-3×FLAG-ProtA::His3MX6, ndc10-1* | This study |
| HZY741 | S288c, MATa, *sml1∆*::*TRP1*, *rad53∆*::*His3MX6*, ura3-52, leu2Δ1, trp1Δ63, his3Δ200, lys2ΔBgl, hom3-10, ade2Δ1, ade8 | This study |

- *HF-SMT3* refers to *6×HIS-3×FLAG-SMT3*. TAF refers to *6×HIS-3×FLAG-Protein A*.

| Plasmid | DESCRIPTION | Source |
| --- | --- | --- |
| HZE2617 | pRS315-*ULP2::kanMX6* | This study |
| HZE2618 | pRS315-*ulp2-SIM^3A^::kanMX6* | This study |
| HZE2619 | pRS315-*ulp2-SIM^3A^CCR^3A^::kanMX6* | This study |
| HZE2620 | pRS315-*ulp2-CCR^3A^::kanMX6* | This study |
| HZE2340 | pRS315-*ULP2-TAF::kanMX6* | de Albuquerque, et al., J Biol Chem 2018 |
| HZE2341 | pRS315-*ulp2-SIM^3A^-TAF::kanMX6* | As above |
| HZE2525 | pRS315-*ulp2-SIM^3A^CCR^3A^-TAF::kanMX6* | As above |
| HZE2527 | pRS315-*ulp2-CCR^3A^-TAF::kanMX6* | As above |
| HZE2557 | LIC-2CT-*ULP1^403-621^-C580S* | This study |
| HZE1809 | LIC-2GT-*ULP2^873-1034^* | This study |
| HZE1810 | LIC-2GT-*ULP2^873-1034^CCR^3A^* | This study |
| HZE2768 | LIC-2GT-*ULP2^873-1034^, Δ(900-902)* | This study |
| HZE2769 | LIC-2GT-*ULP2^873-1034^CCR^3A^, Δ(900-902)* | This study |
| HZE2746 | LIC-2GT-*ULP2^896-1034^* | This study |
| HZE2744 | LIC-2GT-*ULP2^873-979^* | This study |
| HZE2745 | LIC-2GT-*ULP2^873-950^* | This study |
| HZE2732 | pFastBac-LIC-438A-*CTF3-6×HIS-MCM16-MCM22* | This study |

- TAF refers to *6×HIS-3×FLAG-Protein A*.
